# Evidence-based roadside clear-zone design for mitigating run-off-road and rollover crashes on rural highways in Saudi Arabia

**DOI:** 10.1371/journal.pone.0357284

**Published:** 2026-09-16

**Authors:** Turki A. Alamoudi, Saif A. Alarifi

**Affiliations:** Civil Engineering Department, King Saud University, Riyadh, Saudi Arabia; University of Wisconsin-Milwaukee, UNITED STATES OF AMERICA

## Abstract

Saudi Arabia’s rural highway network experiences a high number of severe run-off-road and rollover crashes, yet evidence-based, locally validated roadside clear-zone design guidance remains limited. This study investigates how roadside clear-zone characteristics influence the occurrence and severity of these crashes by integrating statistical analysis of in-depth investigations of multi-fatality crash data with high-fidelity vehicle simulations. An in-depth dataset of 127 multi-fatality crashes (2020–2022) from the National Road Safety Centre was analyzed using binary logistic regression to identify key predictors of run-off-road severity. The final model retained three significant variables: Vehicle Type (odds ratio OR = 0.56 per class increase, p < 0.001), indicating that heavier vehicle categories diminish rollover risk; Posted Speed (OR = 1.017 per km/h increase, p = 0.032), reflecting elevated crash likelihood at higher speed limits; and Steep Slope (V:H ≥ 1:4) (OR = 2.73, p = 0.054), underscoring the destabilizing effect of steep embankments on vehicle stability. Descriptive statistics revealed an average of 3.59 fatalities and 2.80 injuries per crash, with single-vehicle incidents comprising 78% of the sample. Complementary finite-element simulations using AnalyzerPro examined sixteen scenarios that varied clear-zone width (3–7 m), embankment gradient (1:2.5 vs. 1:10), and soil compaction at speeds of 100 km/h and 140 km/h. Widening the clear zone from 3 m to 7 m reduced rollover probability by approximately 45%, while fully compacted embankments and slopes consistently prevented rollovers across all vehicle classes and speed profiles. Steeper slopes markedly increased rollover propensity relative to gentler grades. These findings provide robust, evidence-based guidance for updating roadside design standards—emphasizing clear-zone widening, soil compaction protocols, and slope moderation—to enhance safety on Saudi Arabia’s rural road network.

## Introduction

Traffic safety has become a national priority in Saudi Arabia as the country continues to expand and modernize its transportation infrastructure. With a road network exceeding 73,000 kilometers, managed by the Ministry of Transport and Logistic Services (MoTLS), the Kingdom has made substantial investments in highway development to support economic growth, mobility, and regional connectivity. As part of Vision 2030, various strategic initiatives have been launched to enhance transportation safety, aiming to reduce crash-related fatalities and improve the overall resilience of the road system. Among these efforts is the National Transformation Program [1], which sets a target to lower the road fatality rate to 8 deaths per 100,000 capita by 2030.

Despite these advancements, traffic crashes remain a critical public safety concern, particularly in rural areas. From October 2016 to September 2021, Saudi Arabia recorded approximately 20,362 run-off-road and rollover crashes, accounting for 32.9% of all reported traffic incidents during that period. These crashes resulted in 2,375 fatalities and 15,588 injuries—representing 29.9% and 31.7% of total traffic-related deaths and injuries, respectively. For the purposes of this study, a run-off-road (ROR) crash is defined as a crash in which a vehicle departs the travel lane and encroaches onto the roadside, potentially striking roadside features or traversing roadside terrain. A rollover crash is defined as a crash in which the vehicle rotates onto its side or roof about its longitudinal axis. Although rollover crashes frequently occur following a run-off-road event, they are treated as a separate crash outcome in this study. Such incidents are often severe in nature due to high travel speeds, limited roadside recovery areas, and suboptimal roadside conditions. These statistics underscore the urgent need for targeted safety interventions, particularly those that address the geometric and roadside design factors contributing to crash severity.

**Fig 1:**
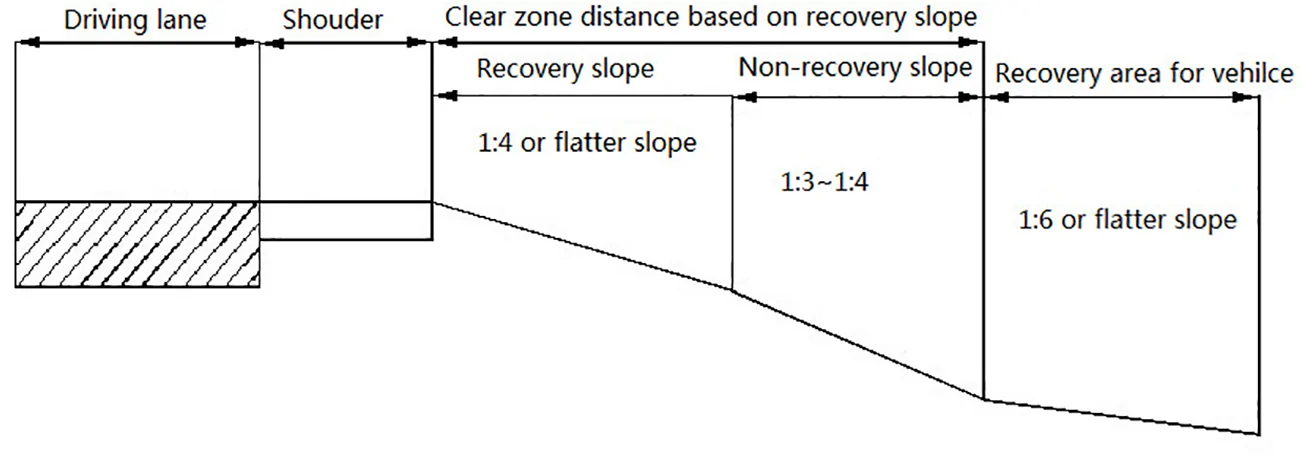
Typical section of a roadside area (Source: 2).

A critical, yet underexplored, aspect of rural highway safety is the design and effectiveness of roadside clear zones. These areas, which extend from the edge of the travel lane and may include the shoulder, recoverable and non-recoverable slopes, and clear run-out spaces, are intended to offer errant vehicles an opportunity to regain control or stop safely. When appropriately designed and maintained, clear zones can significantly reduce the severity of run-off-road and rollover crashes. However, limited research has been conducted—especially in the context of the Gulf region—to evaluate how specific clear zone characteristics influence crash outcomes. As illustrated in Fig 1, a well-designed clear zone must be free of fixed objects and consider factors such as departure speed, vehicle trajectory, and surface conditions [2].

This study aims to assess the safety impact of roadside clear zone conditions on the severity of departure-related crashes in Saudi Arabia. The research is guided by two primary objectives: first, to perform a statistical analysis of multi-fatality crash data from intercity roads from 2020 to 2022, focusing specifically on run-off-road and rollover incidents; and second, to evaluate the influence of key roadside variables—such as slope gradient, surface friction characteristics, pavement edge height, vehicle type, and departure speed and angle—using advanced simulation modeling via Analyzer Pro software. By integrating empirical crash data with simulation-based analysis, the study provides evidence-based insights to inform the development of improved roadside design standards and support data-driven policymaking aimed at reducing crash severity on rural highways.

## Literature review

Safe roadside design has long been recognized as a cornerstone of crash‐severity mitigation. Dumbaugh [3] and Shankar et al. [4] demonstrated that unobstructed, traversable zones adjacent to travel lanes significantly reduce the likelihood and severity of run‐off‐road and rollover incidents by affording drivers space to decelerate or regain control.Building on these early findings, La Torre et al. [5] formalized the concept of a ‘forgiving roadway’ with gently sloped shoulders and clear recoverable areas—designed expressly to accommodate human error. Beyond fixed geometric prescriptions, Roque and Jalayer [6] argued for hazard‑based policies that dynamically adjust clear‑zone width to environmental and traffic fluctuations. Lastly, Stigson et al [7] bring a contemporary view, focusing on rural road designs and how they can be optimized to reduce run-off-road (ROR) crash incidences, considering the unique challenges posed by less urbanized areas.

Parallel to these conceptual advances, design guidance for clear‐zone dimensions and treatments has matured. Both the AASHTO Roadside Design Guide [8] and the Saudi Highway Code [9] prescribe context‑sensitive clear‑zone dimensions—adjusting width, slope, and offset by design speed, traffic volume, and right‑of‑way constraints—while the SHC further mandates specific embankment compaction standards and material selections to account for regional soil and climatic conditions. UNESCAP [10] provides further insights into the nuanced application of clear zone principles in urban and rural settings. It outlines specific measures such as employing breakaway features for street furniture and other non-traversable features to enhance safety in densely populated areas. Lamm et al. [11] and Dupre and Bisson (2006) developed quantitative methods linking vehicle departure trajectories and deceleration needs to clear‑zone width. Cheng et al. [12] then proposed deceleration‑distance‑based criteria, while noting that traffic volume and vehicle mix must also be considered. Empirical studies by Jurewicz and Pyta [13] and Fitzpatrick et al. [14] demonstrate that clear‑zone widths exceeding 8 m, combined with adequately compacted embankments, reduce run‑off‑road crash risk by over 20%. These findings underscore the critical roles of material properties and slope gradients.

A substantial body of work has examined the determinants and severity of roadway departure crashes themselves. Mayora et al. [15] and Jalayer et al. [16] used statistical models to link roadside geometry directly to crash severity and frequency. Spainhour and Mishra [17] and Fitzpatrick et al. [18] further demonstrate that rumble strips and managed vegetation within clear zones enhance driver recovery and reduce crash rates. NHTSA’s [19] nationwide U.S. study and Van Petegem and Wegman’s [20] Dutch analysis both confirm that road curvature, vehicle speed, and visibility jointly determine crash risk. These findings hold true across a variety of roadway and environmental contexts complementing these findings, Khan et al.[21,22] demonstrate that consistent road geometry and rigorous speed management on rural Australian networks further mitigate departure‐related incidents. Table 1 summarizes key empirical studies and their identified predictors of run‑off‑road crashes.

**Table 1:** Summary of key variables for run-off road crashes.

Article	Main Focus	Key Variables Impacting ROR crashes
Duddu et al. [23]	Influence of various factors on single-vehicle crashes	Road alignment, weather conditions, driver behaviour, vehicle type
Spainhour and Mishra [17]	Effectiveness of rumble strips on run-off-road crashes	Presence and type of rumble strips, road geometry, vehicle speed
NHTSA [19]	Fatal single-vehicle ROR crash factors	Road curvature, vehicle speed, environmental conditions, driver intoxication
Dissanayake and Roy [24]	Impact of roadside features on ROR crashes	Roadside features (barriers, clear zones), driver distraction, road surface conditions
Khan et al. [21]	Traffic impact on road crashes	Traffic volume, road surface conditions, time of day, road geometry
Yu and Shen [25]	Roadside infrastructure and driver safety systems	Roadside infrastructure, visibility, signage, driver alert systems
Tomash et al [26]	Role of driver assistance systems in preventing ROR crashes	Advanced driver assistance systems, road design, driver behaviour
Rezapour et al. [27]	Safety impact of roadside barriers and rumble strips	Roadside barriers, road width, shoulder width, rumble strip placement
Fitzpatrick et al. [18]	Influence of clear zones and vegetation on driver behaviour	Clear zone size, roadside vegetation density, road geometry
Van Petegem and Wegman [20]	Dutch road design and ROR crash risk	Traffic volume, road characteristics, safety zone design
Khan et a.l [22]	Consistency of road design and its effects on ROR crashes	Road design consistency, speed limits, road surface, environmental factors
Gong and Fan [28]	Environmental impact and driver fatigue on ROR crashes	Road surface conditions, driver fatigue, traffic density, environmental visibility

Advancements in statistical modeling have enhanced our ability to quantify these relationships. Dissanayake and Roy [24] demonstrate that binary logistic regression effectively pinpoints driver and vehicle factors that worsen crash severity. Van Petegem and Wegman [20] show that negative‑binomial generalized linear models are better suited to overdispersed counts of crash events. Eluru et al. [29] introduced a Mixed Generalized Ordered Response Logit model that permits variable effects to vary by injury severity. Jalayer and Zhou [30] employ nested‑logit methods to further account for heterogeneity in environmental, vehicle, and driver behavior influences. The convergence across these studies lies in their collective goal to better understand the determinants of crash severity through regression analysis.

Crash simulation technologies now offer a powerful complement to field studies. Borovinsek et al. [31] validated finite‐element analyses against full‐scale crash tests, paving the way for detailed kinematic modeling of vehicle–roadside interactions. Ren et al. [32] leveraged tools such as Analyzer Pro to recreate run‐off‐road and rollover dynamics under controlled conditions, while Yang et al. [33]and Astarita and Giofrè [34] incorporated connected‐vehicle data and driver‐error modeling into microsimulation frameworks. These virtual experiments permit systematic evaluation of clear‐zone width, slope compaction, and barrier performance across vehicle classes and speed profiles, offering nuanced insights that are difficult to obtain through observational studies alone.

In conclusion, previous studies have identified individual clear‑zone parameters—slope gradient, surface‑friction properties, pavement‑edge height, and vehicle departure dynamics—as key drivers of run‑off‑road crash severity, yet their combined effects on high‑speed, low‑volume rural highways remain poorly understood. To bridge this gap, the present study merges comprehensive crash data with advanced finite‑element simulations, producing evidence‑based, context‑sensitive roadside design guidelines aimed at significantly improving safety on these vulnerable road networks.

## Data collection and preparation

The dataset analyzed in this study comprises 127 in-depth investigations of multi-fatality crashes, each involving more than two fatalities. For every crash, a specialized investigation team from NRSC conducted a comprehensive site investigation to document roadway geometry, roadside characteristics, vehicle damage, environmental conditions, physical evidence, and other engineering variables relevant to crash reconstruction. These in-depth investigations provide substantially richer information than routine police crash records and form the basis for the statistical and simulation analyses presented in this study. Nevertheless, because the NRSC investigation program focuses on multi-fatality crashes, the dataset represents the most severe end of the crash severity spectrum. Consequently, the estimated relationships primarily reflect factors associated with multi-fatal roadway departure crashes.

A structured dataset was developed from unstructured technical crash investigation reports obtained from the National Road Safety Centre (NRSC). This in-depth dataset focuses on crashes involving multiple fatalities, offering valuable insights into the most severe incidents on Saudi roads. The process involved manually transcribing qualitative narratives into a quantitative format, followed by extensive data cleaning to correct errors, address outliers, and resolve inconsistencies. To ensure accuracy and reliability, validation checks were performed at multiple stages. Each variable was clearly defined and systematically categorized. Preliminary statistical analyses were then conducted to examine regional crash distributions and speed-related crash outcomes. The final dataset comprises 127 crash incidents. Table 2 shows the extracted information for each crash incident.

**Table 2:** Description of variables of the in-depth crash data.

Variable	Type of data	Description
*Crash Date*	Categorical	Year, when the crash occurs
*Night Crashes*	Binary	1 if the crash occurred during night-time
*Region*	Categorical	Administrative Region of Kingdom of Saudi Arabia
*Road Type*	Categorical	Road category
*Run-off Crashes*	Binary	1 if the crash was associated with a Run-off Crash
*No. Of Fatalities*	Numerical	No of Fatalities that occurred
*No of Injuries*	Numerical	No of Injuries that occurred
*No of Vehicles*	Numerical	No of Vehicles involved in the crash
*Vehicle Type*	Categorical	Type of Vehicle involved in the crash
*85 Percent Speed (Kph)*	Numerical	The speed at or below which 85% of the drivers travel on a road segment
*Posted Speed (Kph)*	Numerical	The posted speed limit at the road segment
*No of Lanes*	Numerical	No of lanes per direction at the road segment
*Median Width*	Numerical	The width of the median separating two directions of traffic
*Clear zone width (m)*	Numerical	The unobstructed, traversable roadside are at the side of the road
*Slope (V:H)*	Binary	The gradient of the slope. 1 if it is steeper than or equal to 1:4
*Shoulder drop-off (cm)*	Numerical	Dimension of the road shoulder drop-off

Table 3 summarizes the key descriptive statistics for the 127 crash incidents in the study. On average, each crash resulted in 3.59 fatalities and 2.80 injuries, and involved approximately 1.88 vehicles, underscoring the high-severity nature of these multi‐fatality events, with roughly 42% of crashes occurring at night. Vehicle speeds exhibit considerable variability: 85th percentile speeds range from 50 to 180 km/h, while posted limits span 30–140 km/h. Roadway cross‐section features likewise vary widely—median widths fall below 10.55 m in 75% of cases (and are absent on some segments), whereas clear‑zone widths average 9.62 m but range substantially across sites. This heterogeneity in speed regimes, roadway typologies, and safety‐critical features highlights the diverse environments in which severe run‑off‑road and rollover crashes occur, and underscores the need for context‑sensitive design interventions. Lastly, although both run-off-road and rollover crashes are addressed in this study, the regression model uses run-off-road crash as the dependent variable because this outcome was consistently recorded for all investigated crashes. Rollover was not uniformly documented as a standalone binary variable and therefore could not be reliably modelled using logistic regression. Instead, rollover behavior is examined through the kinematic simulation scenarios.

**Table 3:** Descriptive statistics of in-depth crash data.

Variable	mean	std	min	25%	50%	75%	max
*Crash Date*	2.61	0.94	1	2	3	3	4
*Night Crashes*	0.42	0.50	0	0	0	1	1
*Region*	4.41	2.98	1	1	4	7	13
*Road Type*	1.66	0.78	1	1	1	2	3
*Run-off Crashes* ***(Dependent Variable)***	0.30	0.46	0	0	0	1	1
*No. Of Fatalities*	3.59	1.39	1	3	3	4	9
*No of Injuries*	2.80	5.17	0	0	1	4	47
*No of Vehicles*	1.88	1.01	1	1	2	2	10
*Vehicle Type*	2.99	1.50	1	2	3	5	5
*85 Perc. Speed (Kph)*	118.96	23.56	50	102.75	122	135	180
*Posted Speed (Kph)*	100.47	26.85	30	80	100	120	140
*No of Lanes*	1.80	0.97	1	1	1	3	5
*Median Width*	5.28	6.98	0	0	0	10.55	30
*Clear zone width (m)*	9.62	8.07	0	4.775	8	12	40
*Shoulder drop-off (cm)*	0.56	2.12	0	0	0	0	17

Prior to model estimation, Pearson correlation coefficients were calculated as an initial screening tool to identify pairs of variables exhibiting moderate linear association. This preliminary assessment was intended to reduce the likelihood of introducing highly correlated predictors into the same regression model and was not used as the sole criterion for variable exclusion. Subsequently, multicollinearity among the retained variables was formally evaluated using the Variance Inflation Factor (VIF). All predictors included in the final model exhibited VIF values well below commonly accepted thresholds, confirming that multicollinearity did not materially influence the estimated regression coefficients.

**Fig 2:**
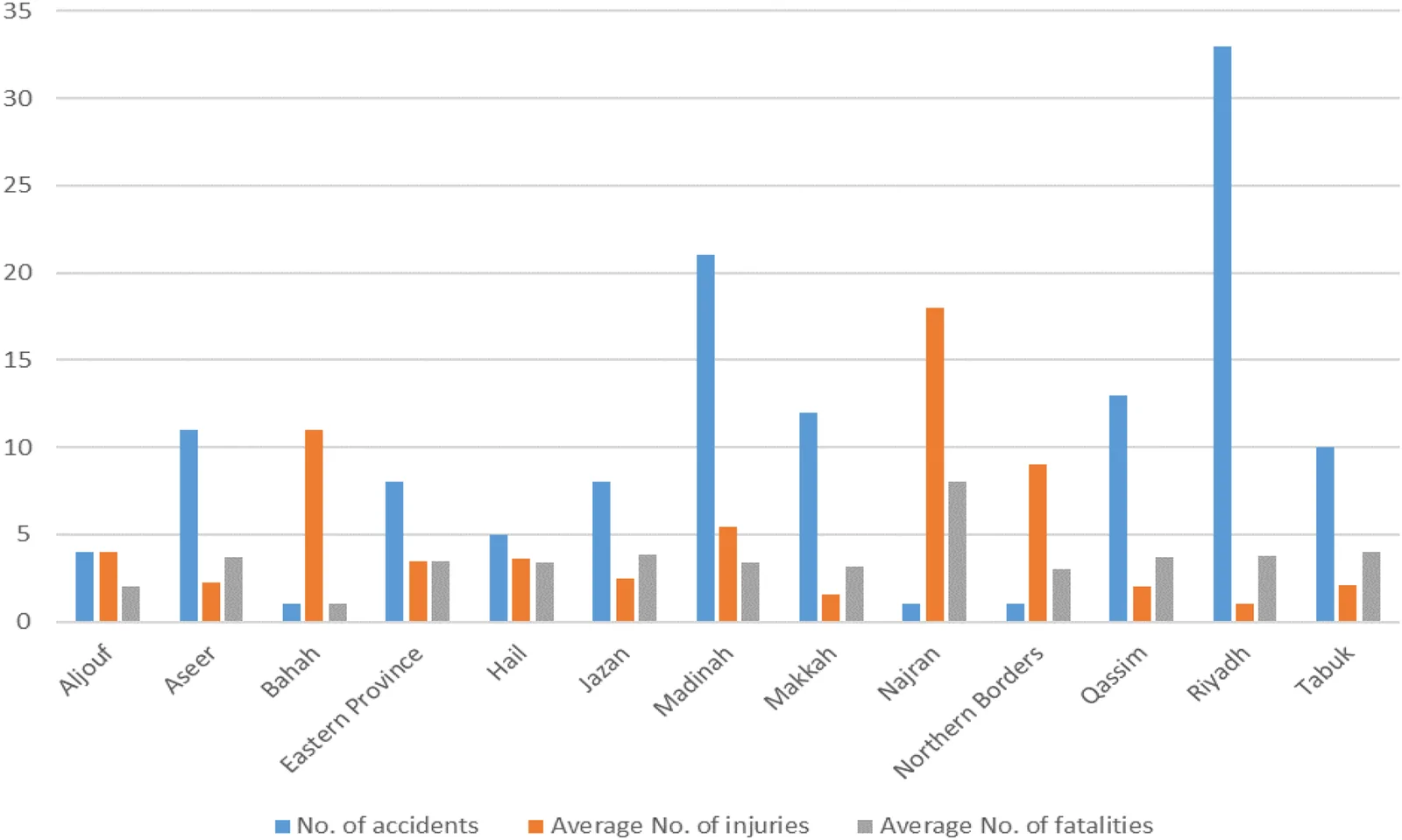
Number of crashes and severity per Region.

In general, the In-Depth Crash dataset reveals a marked disparity in traffic crashes across the regions of Saudi Arabia as shown in Fig 2, with densely populated and urbanized areas like Riyadh experiencing the highest incidence. This disparity suggests a correlation between crash frequency and factors such as traffic volume, urban density, and perhaps the adequacy of road infrastructure. Fig 2 further demonstrates that the investigated crashes are not uniformly distributed across the administrative regions of Saudi Arabia. The Riyadh Region contributes the largest proportion of cases, while other regions are represented to varying degrees. This distribution reflects the characteristics of the NRSC in-depth crash investigation cases rather than a deliberate sampling strategy. Consequently, regional differences in traffic demand, roadway characteristics, and crash exposure should be considered when interpreting the broader applicability of the developed model, although the dataset collectively captures a diverse range of rural highway environments across the Kingdom.

**Fig 3:**
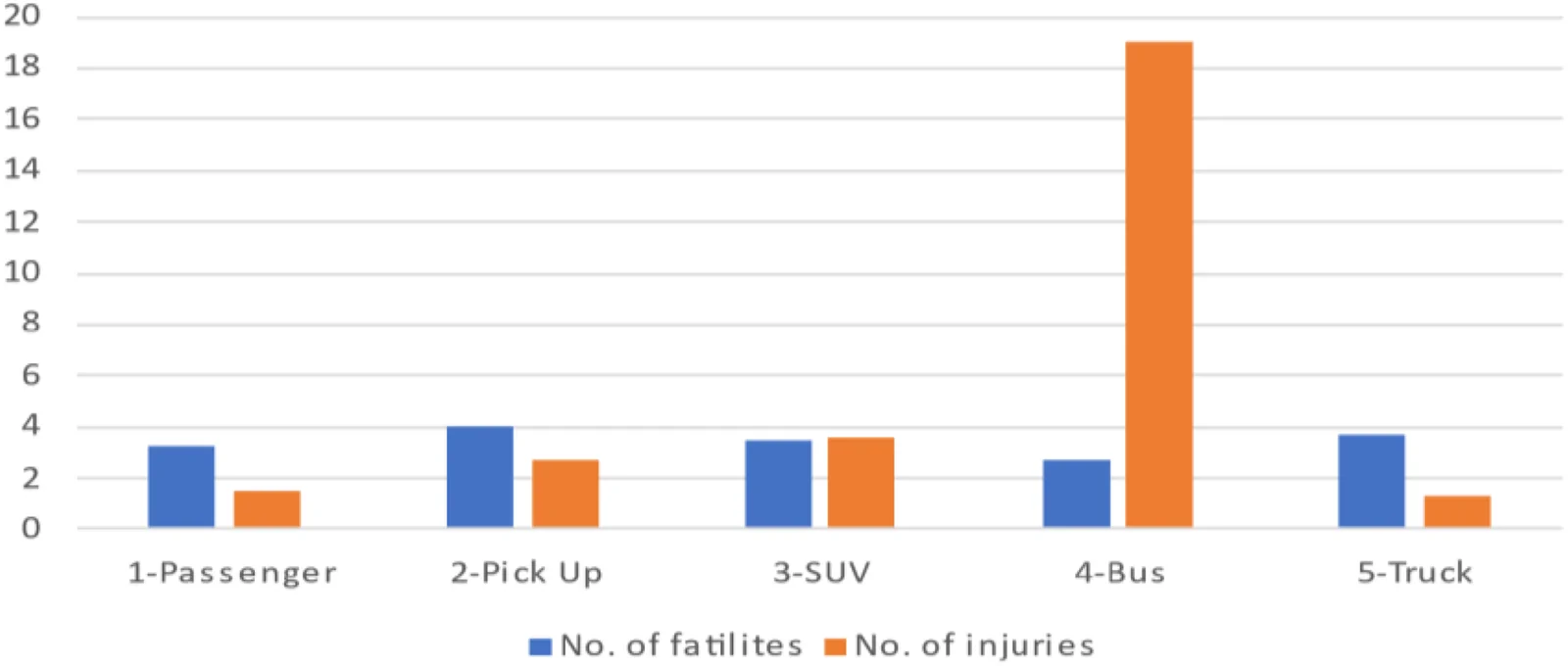
Number of crashes and severity per Vehicle Type.

Fig 3 illustrates the distribution of injuries and fatalities by vehicle type, revealing distinct patterns in crash severity. Analysis of the crash data shows that passenger cars (Vehicle Type 1) are involved in crashes with an average of approximately 3.22 fatalities per incident, along with relatively fewer injuries. Pickups (Vehicle Type 2) exhibit the highest fatality rate, averaging 4 deaths per crash, suggesting a notably higher severity in collisions involving these vehicles. SUVs (Vehicle Type 3) demonstrate a moderate fatality average of 3.51 but are associated with a higher number of injuries, indicating that such crashes frequently result in multiple casualties. Buses (Vehicle Type 4) are particularly significant for their elevated injury rate, averaging 19 injuries per crash, although they are involved in comparatively fewer fatal outcomes. In contrast, trucks (Vehicle Type 5) show a lower average injury count of 1.32 but a relatively high fatality rate of 3.73, underscoring the potentially lethal consequences of crashes involving heavy vehicles. These findings highlight the importance of developing vehicle-specific safety measures and intervention strategies tailored to the unique risk profiles associated with different vehicle types.

**Fig 4:**
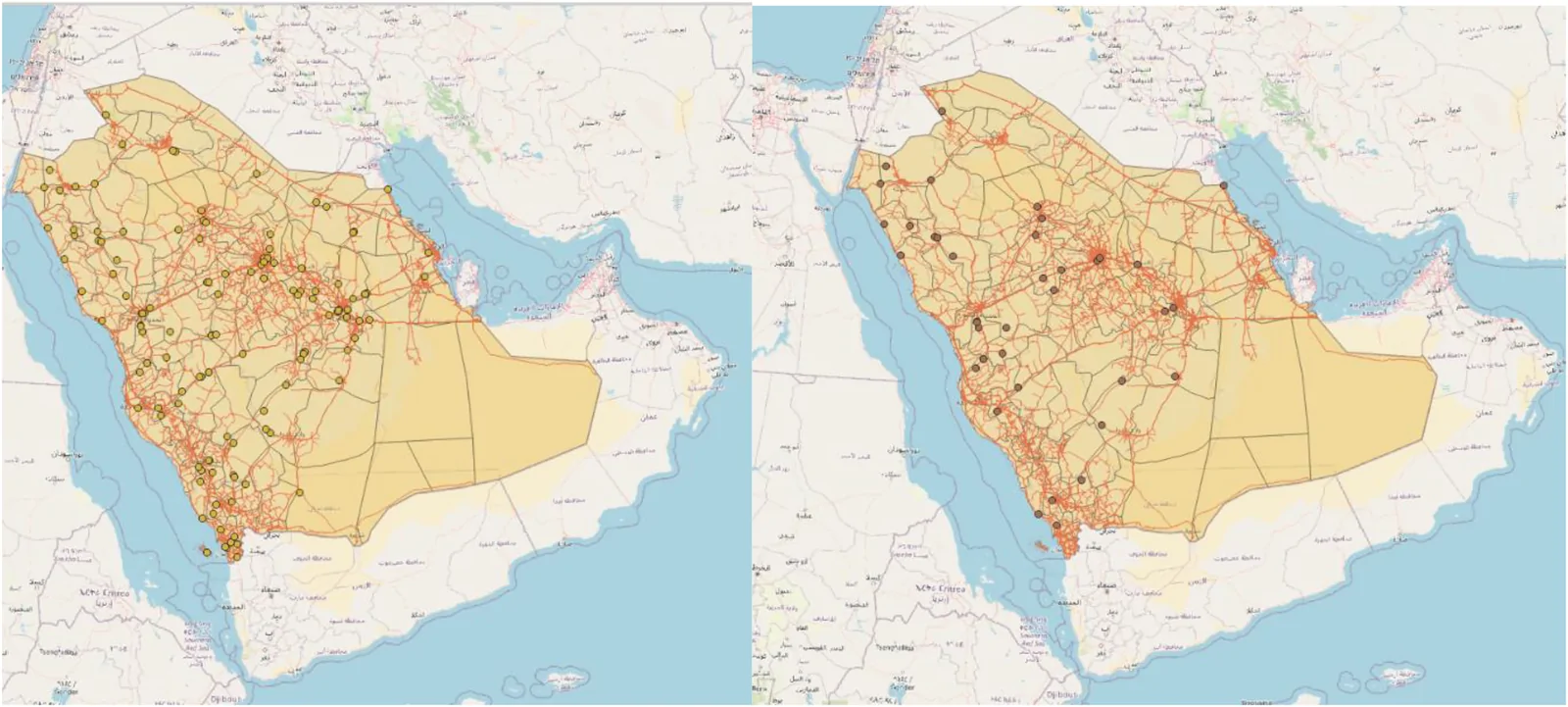
Location of crashes in the NRSC dataset: All crashes (left) and run-off crashes (right).

Fig 4, generated in QGIS, contrasts the spatial patterns of all recorded crashes with those of run‑off‑road incidents. The first map reveals that general crash occurrences are broadly distributed but cluster in urban centers and along primary corridors, reflecting higher traffic volumes. In contrast, the run‑off‑road crash map shows a more discrete pattern: these incidents concentrate on high‑speed roadways and at complex nodes, particularly near interchanges and major junctions. Together, these maps suggest that while overall crash risk correlates with traffic density, run‑off‑road collisions are driven by factors associated with elevated speeds and demanding driving maneuvers.

## Methodology

### Binary logistic regression model

This study follows a structured econometric approach to develop a Binary Logistic Regression (BLR) model tailored to the analysis of run-off road crash data in Saudi Arabia. The binary logistic regression model was developed using the same dataset of 127 in-depth investigated multi-fatality crashes described in Section 3. Of these, 38 crashes (29.7%) were classified as run-off-road crashes (coded as 1), while the remaining 90 crashes (70.3%) represented other crash types (coded as 0). No additional dataset was used for model estimation. The methodology integrates standard model-building procedures with specific adaptations to account for the complex and context-specific characteristics of the NRSC crash dataset. Each step is designed to ensure statistical rigor, reduce bias, and improve interpretability.

Binary logistic regression was selected because the dependent variable is binary and the study seeks to identify interpretable relationships between roadway characteristics and run-off-road crash occurrence. Given the relatively modest sample size and the objective of producing engineering-oriented guidance, logistic regression provides a robust and transparent modelling framework. While machine learning techniques may offer improved predictive performance with larger datasets, they generally require substantially more observations and often provide less interpretable results. Future research using larger crash databases may explore these approaches for comparison

**Fig 5:**
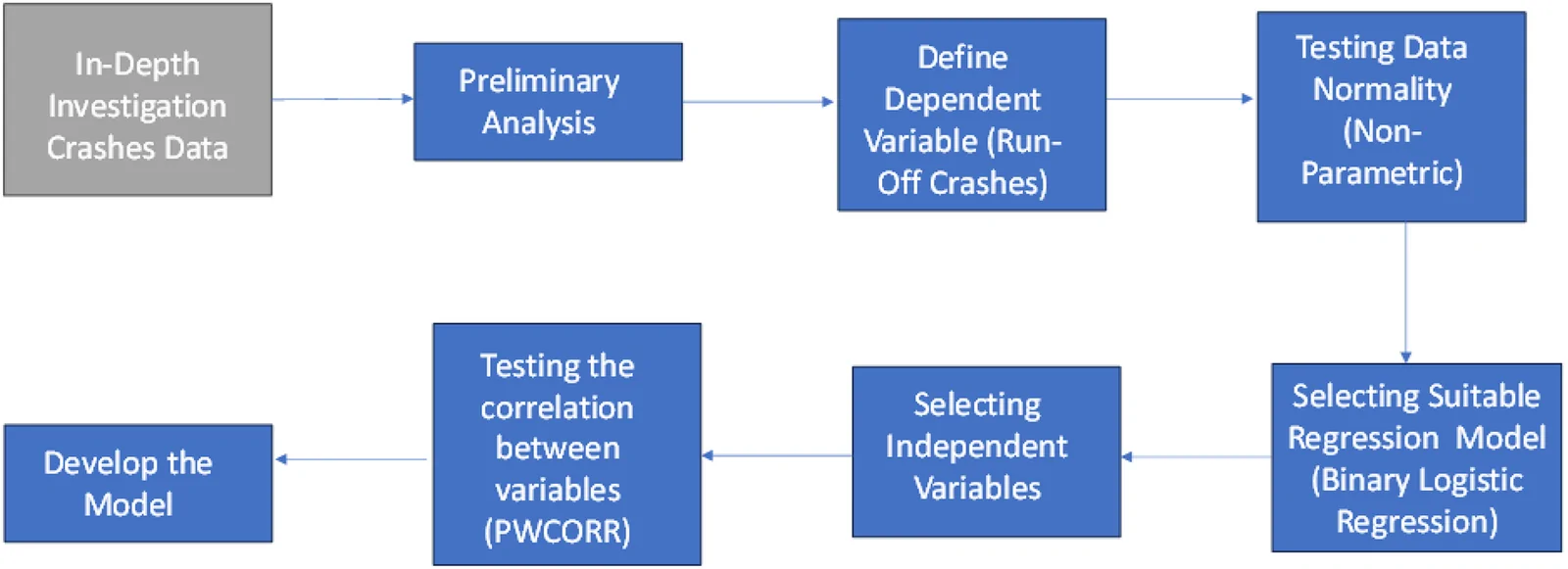
Flowchart of NRSC model specification.

The modeling process, as outlined in Fig 5, involves multiple steps. The flowchart begins by defining the binary dependent variable—run‑off‑road crash (1) versus other crashes (0)—and conducting an initial exploratory analysis to inspect data distributions and identify any need for variable transformation. Given the dichotomous outcome, a Binary Logistic Regression model is then selected. Predictor variables are chosen on the basis of theoretical justification and prior research, and are screened for pairwise associations using Pearson correlations and for multicollinearity via VIF.

Several estimation techniques are available for binary logistic regression, and the choice among them depends on the research design and data characteristics. The most prevalent approach is Maximum Likelihood Estimation (MLE), which derives coefficient estimates by maximizing the likelihood of the observed sample and, under correct model specification, yields asymptotically unbiased and efficient estimates. Alternatively, stepwise procedures—forward selection or backward elimination—can be employed to introduce or remove predictors sequentially based on significance tests, though they require cautious application to avoid overfitting and inflated Type I error rates. In contexts with many covariates or pronounced multicollinearity, penalized regression techniques offer greater stability: Ridge regression (L2 penalty) shrinks coefficient magnitudes by adding a penalty proportional to the squared coefficients, while Lasso regression (L1 penalty) encourages sparsity by imposing a penalty on the absolute values, effectively performing variable selection alongside shrinkage. Finally, when sample sizes are limited or substantive prior knowledge exists, a Bayesian logistic framework may be adopted, specifying priors for each parameter and obtaining posterior distributions—typically via Markov Chain Monte Carlo—that directly incorporate uncertainty.

With the final set of independent variables confirmed, the logistic regression is specified and estimated in SPSS according to the standard form:


$$\begin{array}{c} n\;\frac{P}{\left(\text{1}-P\right)}\;=\;{\beta \;}_{\text{0}}+\;{\beta \;}_{\text{1}}X_{\text{1}}+{\beta \;}_{\text{2}}X_{\text{2}}+\ldots +{\beta \;}_{i}X_{i} \end{array}$$ (1)

Where, P denotes the probability of a run‑off‑road event, β_0_ is the model intercept, β_i_ are the estimated coefficients, and X_i_ are the list of independent variables. Model adequacy and predictive performance are then assessed using standard diagnostics to ensure a robust, interpretable specification for identifying the key drivers of run‑off‑road crashes.

The model’s discriminative performance will be evaluated using the Receiver Operating Characteristic (ROC) curve and its corresponding Area Under the Curve (AUC). The ROC curve plots the True Positive Rate (TPR) against the False Positive Rate (FPR) as the classification threshold varies between 0 and 1, thereby illustrating the trade‑off between sensitivity and specificity. Specifically, for each threshold,


$$\begin{array}{c} \text{True Positive Rate }(\text{TPR}):\;TPR=\frac{TP}{TP+FN} \end{array}$$ (2)

$$\begin{array}{c} \text{False Positive Rate }(\text{FPR}):\;FPR=\frac{FP}{FP+TN} \end{array}$$ (3)

Where, TP and TN denote correctly predicted positives and negatives, respectively, and FP and FN denote incorrect predictions. The AUC—ranging from 0.5 (no discrimination) to 1.0 (perfect discrimination)—provides a single scalar metric summarizing the model’s overall ability to distinguish between run‑off‑road crashes and other events. A higher AUC indicates superior predictive accuracy and robustness across all possible thresholds.

### Simulation modeling

To complement the statistical analysis of run‑off‑road crashes, a series of high‑fidelity kinematic simulations were conducted in AnalyzerPro to identify the specific road–vehicle configurations most likely to precipitate rollovers. Sixteen representative scenarios were developed based on the detailed crash characteristics extracted from the NRSC dataset. Each scenario systematically varies key parameters—vehicle class, road classification, travel speed, departure angle, vehicle loading, embankment height relative to the clear zone, slope gradient, and soil compaction in both the embankment and adjacent clear zone.

**Fig 6:**
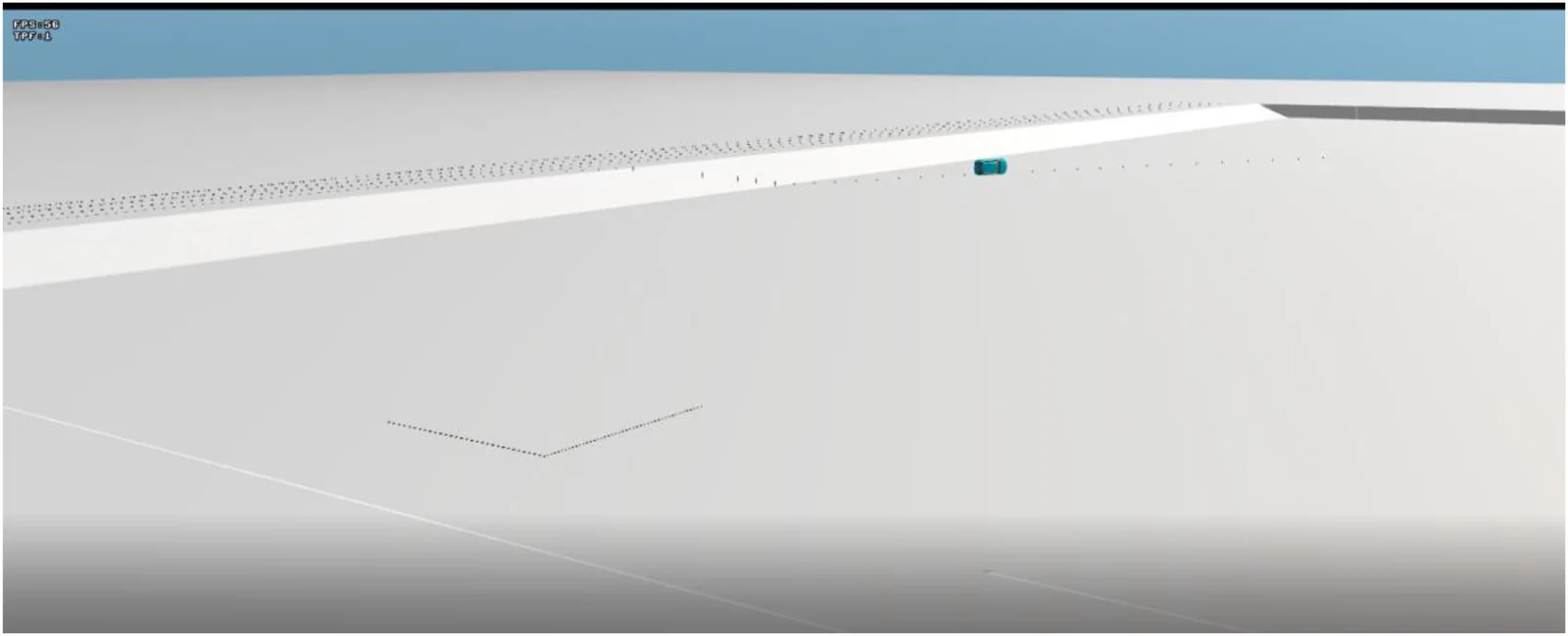
Illustration of the simulation environment of a run-off road incident of a vehicle where rollover takes place.

Each simulation faithfully replicated key geometric features—lane width, horizontal curvature, pavement surface texture, and embankment profile—ensuring that the virtual environment closely matched observed crash sites (Fig 6). Simulator inputs were defined from the processed NRSC data, and vehicle models were configured to standard mass and inertial properties for each class. During each run, AnalyzerPro recorded vehicle trajectory, rollover initiation point, and dynamic metrics such as lateral and vertical accelerations, yaw rate, and post‑departure attitude.

This protocol enables a structured exploration of interactions between roadway design and vehicle behavior under run‑off‑road conditions. The systematic variation of parameters and comprehensive data capture provide a methodological basis for identifying the combinations of factors that precipitate rollover events, thereby informing evidence‑based roadside design and safety countermeasures.

## Analyses and results

### Binary logistic regression modeling results

The forward stepwise logistic regression approach was employed to systematically evaluate the individual and combined contributions of candidate predictors. The final model, which retained ‘Vehicle Type’, ‘Slope (V:H)’, and ‘Posted Speed’ as significant predictors, is presented in Table 4. Across the model-building process, improvements in model fit were evident; the −2 Log Likelihood statistic decreased from 140.703 in Step 1 to 132.073 in Step 3, indicating better alignment between the predicted and observed outcomes with each added variable.

**Table 4:** Variable estimation of the BLR model and the NRSC dataset.

Step	Variable	B	S.E.	Wald	df	Sig.	Exp(B)
**Step 1ᵃ**	Vehicle Type	−0.579	0.155	13.925	1	0.000	0.560
	Constant	0.763	0.439	3.017	1	0.082	2.145
**Step 2ᵇ**	Posted Speed (Kph)	0.017	0.008	4.622	1	0.032	1.017
	Vehicle Type	−0.637	0.164	15.137	1	0.000	0.529
	Constant	−0.836	0.867	0.930	1	0.335	0.433
**Step 3ᶜ**	Posted Speed (Kph)	0.018	0.008	4.921	1	0.027	1.019
	Vehicle Type	−0.648	0.169	14.783	1	0.000	0.523
	slope (V:H)	1.004	0.522	3.706	1	0.054	2.730
	Constant	−1.127	0.905	1.552	1	0.213	0.324

**^a^**Variable(s) entered on step 1: Vehicle Type.

**^b^**Variable(s) entered on step 2: Posted Speed (Kph).

**^c^**Variable(s) entered on step 3: slope (V:H).

An evaluation of the statistical significance of model parameters provides a deeper understanding of how specific predictors influence the likelihood of run-off crashes. *Vehicle Type* emerged as a consistently significant factor throughout all stages of the model development. The negative coefficient of −0.579 (p < 0.001) indicates that higher vehicle classification values are associated with a substantial reduction in the odds of a run-off crash, specifically by a factor of 0.560. This highlights the critical role vehicle characteristics play in influencing crash risk. *Posted Speed (kph)* became significant upon entry into the model, with a positive coefficient of 0.017 (p = 0.032), suggesting that increases in posted speed are associated with higher crash likelihood. Specifically, each additional kilometer per hour in posted speed raises the odds of a run-off crash by a factor of 1.017. This finding is consistent with the broader literature, which links higher speeds to reduced driver reaction time, increased braking distances, and more severe collision outcomes. *Slope (V:H)*, while only marginally significant (coefficient: 1.004, p = 0.054), contributes further insight into crash dynamics. The corresponding odds ratio of 2.730 suggests that steeper slopes may considerably elevate the likelihood of a run-off event, potentially due to their influence on vehicle stability and driver control. Collectively, these predictors reflect the complex interplay of roadway, vehicle, and environmental factors in shaping crash outcomes and offer important implications for targeted safety interventions and infrastructure design strategies.

**Fig 7:**
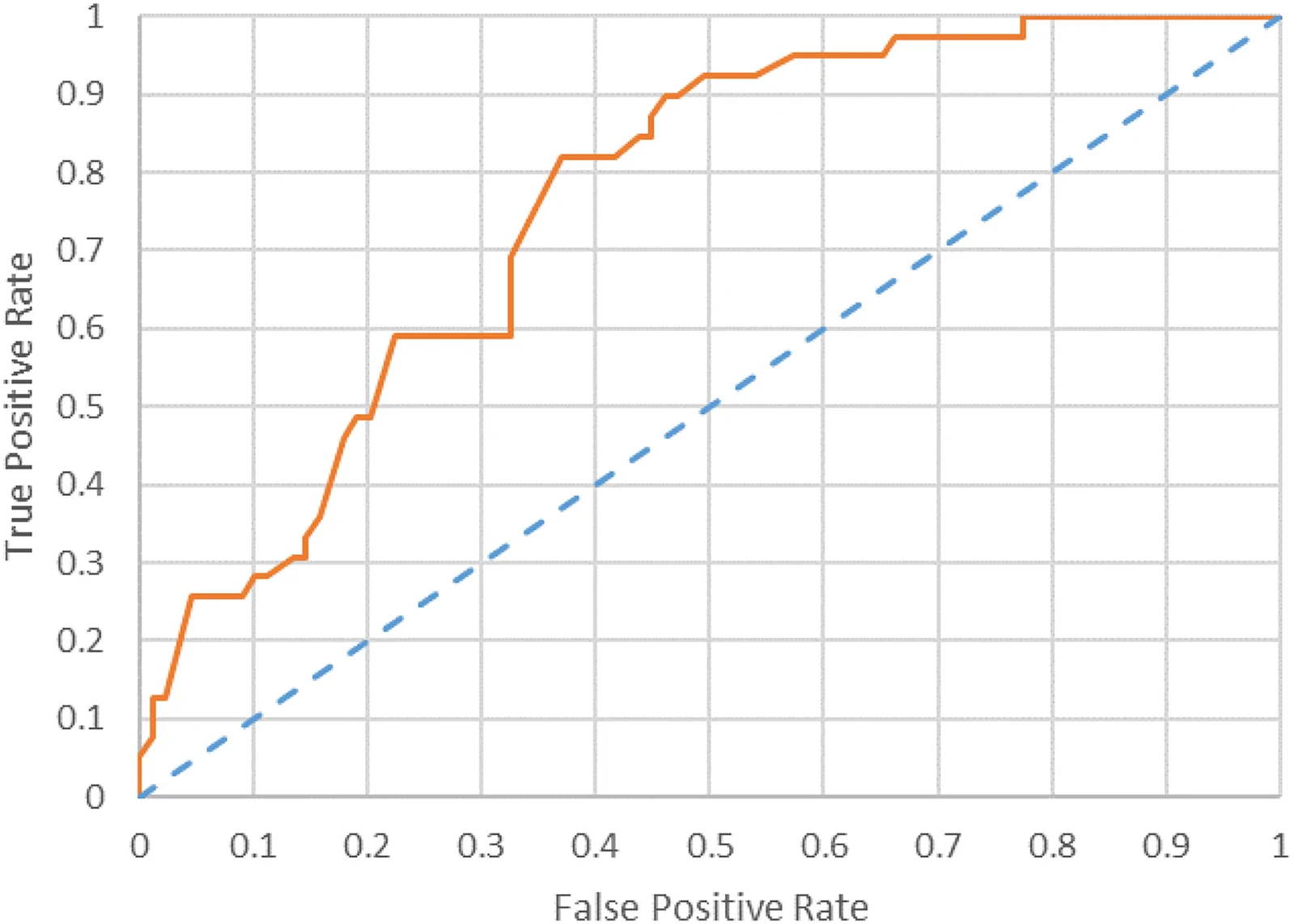
ROC Curve representation.

The Receiver Operating Characteristic (ROC) curve, presented in Fig 7, illustrates the classification performance of the developed logistic regression model. The Area Under the Curve (AUC) is approximately 0.76, indicating a satisfactory level of discriminatory power in distinguishing between run-off and non-run-off crashes. An AUC value of 0.76 suggests that the model correctly differentiates between the two outcomes 76% of the time, which is notably better than random classification (AUC = 0.5) but below the threshold for excellent discrimination (AUC ≥ 0.9). This result confirms that the model—based on the predictors *Vehicle Type*, *Posted Speed (kph)*, and *Slope (V:H)*—demonstrates a reliable capacity to identify crash types, reinforcing the relevance and utility of the selected variables in capturing the underlying crash dynamics.

### Kinematic simulation of run-off crashes

**Fig 8:**
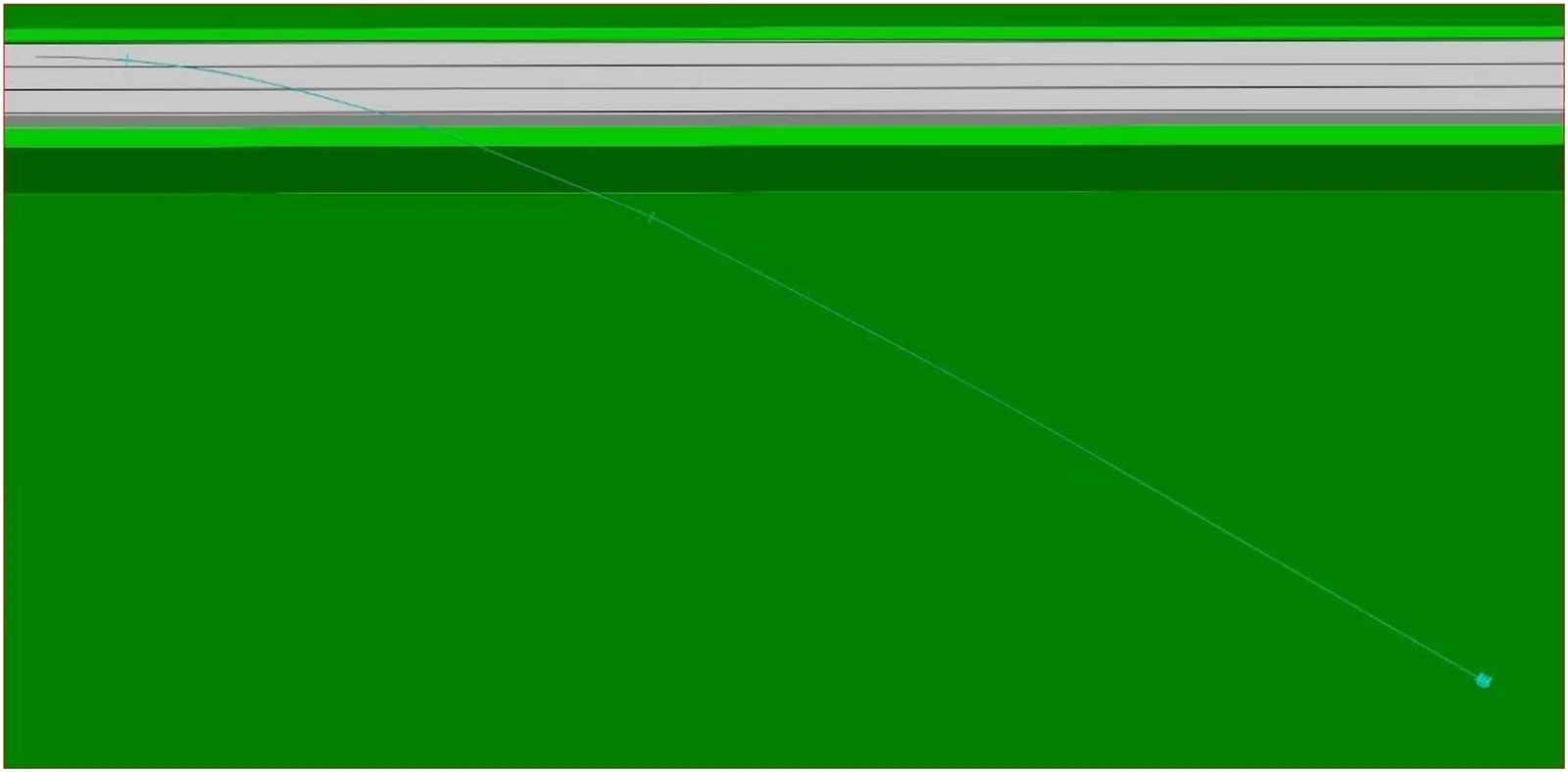
The complete trajectory of a run-off road event with an effective rollover.

The use of AnalyzerPro enables precise parameter adjustments and real-time simulation of vehicle behavior during run-off road crashes. This methodological approach involves systematically testing each scenario while capturing detailed data on vehicle trajectory, rollover initiation points, and the sequence of events leading to loss of control, as depicted in Fig 8. The resulting dataset offers a comprehensive insight into the dynamic interactions between vehicle characteristics, road geometry, and environmental conditions. These high-fidelity simulations enhance the understanding of how specific configurations contribute to rollover risk during critical crash moments.

The scenarios configured, vary the following parameters:

- Vehicle Type: Large SUV (BMW X5 5.0i), Private Car (AUDI A6)
- Road Type: HIGHWAY, ARTERIAL
- Speed (Kph): 100, 140
- Height of Road from Clear Zone (m): 1, 3
- Slope Gradient (1:x): 2.5, 10
- Compaction of Embankment: Compacted, Loose
- Compaction of Clear Zone: Compacted, Loose

In all the scenarios, the following parameters remain constant:

- Angle of Departure: 20^o^
- Load: Full Load

The analysis of vehicular rollover incidents during run-off road scenarios, as summarized in Table 5, underscores the significant influence of embankment and roadside surface conditions on vehicle stability. In particular, the level of soil compaction in both the embankment and the adjacent clear zone emerged as a decisive factor. Simulations conducted under ‘Loose’ compaction conditions for both zones consistently led to vehicle rollovers across multiple vehicle types, including SUVs and passenger cars such as the BMW X5 and Audi A6. These results highlight the critical role that inadequate soil stabilization plays in increasing rollover risk during roadway departures. In contrast, scenarios characterized by ‘Compacted’ soil conditions generally did not result in rollovers, indicating that enhanced ground firmness contributes to improved lateral stability and significantly reduces the likelihood of vehicle overturning under similar circumstances. This finding reinforces the importance of proper roadside and embankment treatment in road safety design and maintenance.

**Table 5:** Summary of the configuration and the result for each scenario of the kinematic simulation.

Sc	Vehicle Type	Road Type	Speed (Kph)	Height from Clear Zone	Slope Grad.	Compaction of Embankment	Compaction of Clear Zone	Result
1	Large SUV	HIGHWAY	140	3	2.5	Compacted	Compacted	No Rollover
2	Large SUV	HIGHWAY	140	3	2.5	Loose	Loose	Rollover
3	Large SUV	ARTERIAL	100	3	2.5	Compacted	Compacted	No Rollover
4	Large SUV	ARTERIAL	100	3	2.5	Loose	Loose	Rollover
5	Large SUV	HIGHWAY	140	1	10	Compacted	Compacted	No Rollover
6	Large SUV	HIGHWAY	140	1	10	Loose	Loose	Rollover
7	Large SUV	ARTERIAL	100	1	10	Compacted	Compacted	No Rollover
8	Large SUV	ARTERIAL	100	1	10	Loose	Loose	Rollover
9	Private Car	HIGHWAY	140	3	2.5	Compacted	Compacted	No Rollover
10	Private Car	HIGHWAY	140	3	2.5	Loose	Loose	Rollover
11	Private Car	ARTERIAL	100	3	2.5	Compacted	Compacted	No Rollover
12	Private Car	ARTERIAL	100	3	2.5	Loose	Loose	Rollover
13	Private Car	HIGHWAY	140	1	10	Compacted	Compacted	No Rollover
14	Private Car	HIGHWAY	140	1	10	Loose	Loose	Rollover
15	Private Car	ARTERIAL	100	1	10	Compacted	Compacted	No Rollover
16	Private Car	ARTERIAL	100	1	10	Loose	Loose	Rollover

Moreover, the embankment gradient at the point of roadway departure plays a pivotal role in rollover dynamics. Vehicles traversing steeper slopes (e.g., 1:2.5) exhibited a markedly higher propensity for rollover compared to those encountering more gradual inclines (e.g., 1:10). This observation underscores the critical influence of slope steepness on the lateral forces acting upon a vehicle during a run-off event. Steeper slopes tend to destabilize the vehicle’s center of gravity more abruptly, thereby increasing the likelihood of rollover, particularly under certain speed and trajectory conditions.

## Conclusion

This study has integrated rigorous statistical modeling with high-fidelity kinematic simulations to elucidate the influence of roadside clear-zone characteristics and embankment conditions on the severity of run-off-road crashes in Saudi Arabia. The binary logistic regression analysis of the 2020–2022 NRSC dataset identified *Vehicle Type*, *Posted Speed*, and *Slope (V:H)* as significant predictors: heavier vehicle classes reduced rollover odds, higher posted limits increased crash likelihood, and steeper embankment slopes elevated rollover risk. The model’s satisfactory discriminative performance (AUC ≈ 0.76) confirms that these variables reliably distinguish between run-off and non-run-off events

Complementing the regression results, the AnalyzerPro simulations—spanning sixteen scenarios that varied vehicle class, speed, departure angle, embankment slope, and soil compaction—demonstrated that soil stability is paramount. Loose compaction in both embankment and clear-zone materials consistently precipitated rollovers across SUVs and sedans, whereas compacted conditions virtually eliminated them. Moreover, steeper slopes (1:2.5) were shown to markedly amplify lateral destabilizing forces compared to gentler grades (1:10), underscoring the need for context-sensitive slope design.

Despite these insights, several limitations warrant consideration. The sample was restricted to 127 multi-fatality crashes, which may limit generalizability to lower-severity incidents. Transcription of narrative reports into structured data introduces potential human error, and simulation models necessarily simplify real-world variability in vehicle designs and environmental factors. Moreover, the cross-sectional nature of the analysis precludes causal inference regarding temporal trends or policy interventions.

The findings of this study are primarily applicable to high-speed, multi-lane divided rural highways with posted speed limits between 100 and 140 km/h, which represent the roadway environments examined in both the in-depth crash dataset and the kinematic simulation scenarios. Based on the combined results of the statistical analysis and vehicle dynamics simulations, wider clear zones, flatter embankment slopes where feasible, appropriate soil compaction, and effective speed management were consistently associated with improved roadside safety performance. However, because the regression analysis is observational and the simulation scenarios represent a defined set of roadway and vehicle conditions, these findings should not be interpreted as definitive evidence of causality. Instead, they should be considered practical recommendations that warrant further validation using larger and more diverse crash datasets, additional roadway environments, and broader simulation scenarios before widespread implementation.

## Supporting information

S1 Data In-depth crash invistigation data correlation -V02. (XLSX)
