# Supplementary material for: Evidence-based roadside clear-zone design for mitigating run-off-road and rollover crashes on rural highways in Saudi Arabia
Source: PLoS One. 2026 Sep 16;21(9):e0357284. doi: 10.1371/journal.pone.0357284 (PMC13581012; doi:10.1371/journal.pone.0357284)
Supplement: Supplementary file 1 [file pone.0357284.s003.docx]

**Response to Reviewers**

**Manuscript ID:** PONE-D-26-08573

**Title**: Evidence-Based Roadside Clear-Zone Design for Mitigating Run-Off-Road and Rollover Crashes on Rural Highways in Saudi Arabia

**Journal**: PLOS ONE

**Corresponding Author:** Dr. Saif Alarifi

**Dear Dr. Iman Aghayan,**

We sincerely thank the reviewers for their thorough and constructive feedback on our manuscript. We have carefully addressed all comments and revised the manuscript accordingly. Below, we provide a point-by-point response to each comment, indicating where changes have been made in the revised manuscript.

**Journal requirements**

When submitting your revision, we need you to address these additional requirements.

**Requirement 1:**

Please ensure that your manuscript meets PLOS ONE's style requirements, including those for file naming. The PLOS ONE style templates can be found at

[https://journals.plos.org/plosone/s/file?id=wjVg/PLOSOne_formatting_sample_main_body.pdf](https://track.editorialmanager.com/CL0/https:%2F%2Fjournals.plos.org%2Fplosone%2Fs%2Ffile%3Fid=wjVg%2FPLOSOne_formatting_sample_main_body.pdf/1/010f019e29865dbe-902774bc-9d7d-4da2-8062-6068bc94f825-000000/oEgdA-dA_wstbELwrxaz6StnX6UI645e3Wf10ms0lU4=258) and [https://journals.plos.org/plosone/s/file?id=ba62/PLOSOne_formatting_sample_title_authors_affiliations.pdf](https://track.editorialmanager.com/CL0/https:%2F%2Fjournals.plos.org%2Fplosone%2Fs%2Ffile%3Fid=ba62%2FPLOSOne_formatting_sample_title_authors_affiliations.pdf/1/010f019e29865dbe-902774bc-9d7d-4da2-8062-6068bc94f825-000000/8HDHTO1DvPg3Y4A_S4pwJ-29quloxMFma9rxPM02keE=258)

**Response 1:**

We thank the Editorial Office for this observation. The manuscript has been revised to fully comply with the PLOS ONE formatting guidelines, including the prescribed manuscript structure, headings, tables, figures, references, and file naming conventions.

**Requirement 2:**

Please note that PLOS One has specific guidelines on code sharing for submissions in which author-generated code underpins the findings in the manuscript. In these cases, we expect all author-generated code to be made available without restrictions upon publication of the work. Please review our guidelines at https://journals.plos.org/plosone/s/materials-and-software-sharing#loc-sharing-code and ensure that your code is shared in a way that follows best practice and facilitates reproducibility and reuse.

**Response 2:**

This study did not involve the development of any author-generated software or custom computer code. Statistical analyses were performed using IBM SPSS Statistics, geographic visualization was prepared using QGIS, and vehicle simulations were conducted using the commercial software AnalyzerPro. Therefore, no custom code underpins the findings of this study.

**Requirement 3:**

We note that you have indicated that there are restrictions to data sharing for this study. PLOS only allows data to be available upon request if there are legal or ethical restrictions on sharing data publicly. For more information on unacceptable data access restrictions, please see http://journals.plos.org/plosone/s/data-availability#loc-unacceptable-data-access-restrictions.

Before we proceed with your manuscript, please address the following prompts:

a) If there are ethical or legal restrictions on sharing a de-identified data set, please explain them in detail (e.g., data contain potentially identifying or sensitive patient information, data are owned by a third-party organization, etc.) and who has imposed them (e.g., a Research Ethics Committee or Institutional Review Board, etc.). Please also provide contact information for a data access committee, ethics committee, or other institutional body to which data requests may be sent.

b) If there are no restrictions, please upload the minimal anonymized data set necessary to replicate your study findings to a stable, public repository and provide us with the relevant URLs, DOIs, or accession numbers. For a list of recommended repositories, please see

https://journals.plos.org/plosone/s/recommended-repositories. You also have the option of uploading the data as Supporting Information files, but we would recommend depositing data directly to a data repository if possible.

We will update your Data Availability statement on your behalf to reflect the information you provide.

**Response 3:**

We thank the Editorial Office for this important request. The dataset used in this study is shared "All data are in the supporting information files" as an excel sheet

**Requirement 4:**

4. Please provide a complete Data Availability Statement in the submission form, ensuring you include all necessary access information or a reason for why you are unable to make your data freely accessible. If your research concerns only data provided within your submission, please write "All data are in the manuscript and/or supporting information files" as your Data Availability Statement.

**Response 4:**

We thank the Editorial Office for this request. The Data Availability Statement has been revised as requested.

**Requirement 5:**

Thank you for stating the following in the Acknowledgments Section of your manuscript:

The authors would like to thank the National Transport Safety Center for providing the crash datasets and for helping in our understanding of the datasets. In addition, the authors would like to thank the Ongoing Research Funding Program, (ORFFT-2025-081-3), King Saud University, Riyadh, Saudi Arabia for financial support. Lastly, we would like to also thank Konstantia Kontodimou for her help.

We note that you have provided funding information that is not currently declared in your Funding Statement. However, funding information should not appear in the Acknowledgments section or other areas of your manuscript. We will only publish funding information present in the Funding Statement section of the online submission form.

Please remove any funding-related text from the manuscript and let us know how you would like to update your Funding Statement. Currently, your Funding Statement reads as follows:

The author(s) received no specific funding for this work.

Please include your amended statements within your cover letter; we will change the online submission form on your behalf.

**Response 5:**

We thank the Editorial Office for identifying this inconsistency. The funding information has been removed from the Acknowledgments section and transferred to the Funding Statement. The Funding Statement has been updated to accurately reflect the financial support received for this study.

**Requirement 6:**

Please update your submission to use the PLOS LaTeX template. The template and more information on our requirements for LaTeX submissions can be found at http://journals.plos.org/plosone/s/latex.

**Response 6:**

We thank the Editorial Office for this note. This requirement is not applicable, as the manuscript was prepared and submitted using Microsoft Word rather than LaTeX. The revised manuscript has been prepared in accordance with the PLOS ONE Microsoft Word formatting guidelines.

**Requirement 7:**

Please amend your authorship list in your manuscript file to include author Saif A. Alarifi.

**Response 7:**

Done

**Requirement 8:**

Please amend the manuscript submission data (via Edit Submission) to include author Saif Alarifi.

**Response 8:**

Done

**Requirement 9:**

[We note that Figure 4 in your submission contain [map/satellite] images which may be copyrighted. All PLOS content is published under the Creative Commons Attribution License (CC BY 4.0), which means that the manuscript, images, and Supporting Information files will be freely available online, and any third party is permitted to access, download, copy, distribute, and use these materials in any way, even commercially, with proper attribution. For these reasons, we cannot publish previously copyrighted maps or satellite images created using proprietary data, such as Google software (Google Maps, Street View, and Earth). For more information, see our copyright guidelines: http://journals.plos.org/plosone/s/licenses-and-copyright.](http://track.editorialmanager.com/CL0/We%2520note%2520that%2520Figure%25204%2520in%2520your%2520submission%2520contain%2520%255bmap%2Fsatellite%255d%2520images%2520which%2520may%2520be%2520copyrighted.%2520All%2520PLOS%2520content%2520is%2520published%2520under%2520the%2520Creative%2520Commons%2520Attribution%2520License%2520(CC%2520BY%25204.0),%2520which%2520means%2520that%2520the%2520manuscript,%2520images,%2520and%2520Supporting%2520Information%2520files%2520will%2520be%2520freely%2520available%2520online,%2520and%2520any%2520third%2520party%2520is%2520permitted%2520to%2520access,%2520download,%2520copy,%2520distribute,%2520and%2520use%2520these%2520materials%2520in%2520any%2520way,%2520even%2520commercially,%2520with%2520proper%2520attribution.%2520For%2520these%2520reasons,%2520we%2520cannot%2520publish%2520previously%2520copyrighted%2520maps%2520or%2520satellite%2520images%2520created%2520using%2520proprietary%2520data,%2520such%2520as%2520Google%2520software%2520(Google%2520Maps,%2520Street%2520View,%2520and%2520Earth).%2520For%2520more%2520information,%2520see%2520our%2520copyright%2520guidelines:%2520http:%2Fjournals.plos.org%2Fplosone%2Fs%2Flicenses-and-copyright.%250b%250bWe%2520require%2520you%2520to%2520either%2520(1)%2520present%2520written%2520permission%2520from%2520the%2520copyright%2520holder%2520to%2520publish%2520these%2520figures%2520specifically%2520under%2520the%2520CC%2520BY%25204.0%2520license,%2520or%2520(2)%2520remove%2520the%2520figures%2520from%2520your%2520submission:%250b%250ba.%2520You%2520may%2520seek%2520permission%2520from%2520the%2520original%2520copyright%2520holder%2520of%2520Figure%25204%2520to%2520publish%2520the%2520content%2520specifically%2520under%2520the%2520CC%2520BY%25204.0%2520license.%2520%250b%250bWe%2520recommend%2520that%2520you%2520contact%2520the%2520original%2520copyright%2520holder%2520with%2520the%2520Content%2520Permission%2520Form%2520(http:%2Fjournals.plos.org%2Fplosone%2Fs%2Ffile%3Fid=7c09%2Fcontent-permission-form.pdf)%2520and%2520the%2520following%2520text:%250b%E2%80%9CI%2520request%2520permission%2520for%2520the%2520open-access%2520journal%2520PLOS%2520ONE%2520to%2520publish%2520XXX%2520under%2520the%2520Creative%2520Commons%2520Attribution%2520License%2520(CCAL)%2520CC%2520BY%25204.0%2520(http:%2F%2Fcreativecommons.org%2Flicenses%2Fby%2F4.0%2F).%2520Please%2520be%2520aware%2520that%2520this%2520license%2520allows%2520unrestricted%2520use%2520and%2520distribution,%2520even%2520commercially,%2520by%2520third%2520parties.%2520Please%2520reply%2520and%2520provide%2520explicit%2520written%2520permission%2520to%2520publish%2520XXX%2520under%2520a%2520CC%2520BY%2520license%2520and%2520complete%2520the%2520attached%2520form.%E2%80%9D%250b%250bPlease%2520upload%2520the%2520completed%2520Content%2520Permission%2520Form%2520or%2520other%2520proof%2520of%2520granted%2520permissions%2520as%2520an%2520%2522Other%2522%2520file%2520with%2520your%2520submission.%250b%250bIn%2520the%2520figure%2520caption%2520of%2520the%2520copyrighted%2520figure,%2520please%2520include%2520the%2520following%2520text:%2520%E2%80%9CReprinted%2520from%2520%255bref%255d%2520under%2520a%2520CC%2520BY%2520license,%2520with%2520permission%2520from%2520%255bname%2520of%2520publisher%255d,%2520original%2520copyright%2520%255boriginal%2520copyright%2520year%255d.%E2%80%9D%250b%250bb.%2520If%2520you%2520are%2520unable%2520to%2520obtain%2520permiss/1/010f019e29865dbe-902774bc-9d7d-4da2-8062-6068bc94f825-000000/PNnKSUZsy__Zcl4IdOUTkaqPrlHmA14iOezaA2RB794=258)

[We require you to either (1) present written permission from the copyright holder to publish these figures specifically under the CC BY 4.0 license, or (2) remove the figures from your submission:](http://track.editorialmanager.com/CL0/We%2520note%2520that%2520Figure%25204%2520in%2520your%2520submission%2520contain%2520%255bmap%2Fsatellite%255d%2520images%2520which%2520may%2520be%2520copyrighted.%2520All%2520PLOS%2520content%2520is%2520published%2520under%2520the%2520Creative%2520Commons%2520Attribution%2520License%2520(CC%2520BY%25204.0),%2520which%2520means%2520that%2520the%2520manuscript,%2520images,%2520and%2520Supporting%2520Information%2520files%2520will%2520be%2520freely%2520available%2520online,%2520and%2520any%2520third%2520party%2520is%2520permitted%2520to%2520access,%2520download,%2520copy,%2520distribute,%2520and%2520use%2520these%2520materials%2520in%2520any%2520way,%2520even%2520commercially,%2520with%2520proper%2520attribution.%2520For%2520these%2520reasons,%2520we%2520cannot%2520publish%2520previously%2520copyrighted%2520maps%2520or%2520satellite%2520images%2520created%2520using%2520proprietary%2520data,%2520such%2520as%2520Google%2520software%2520(Google%2520Maps,%2520Street%2520View,%2520and%2520Earth).%2520For%2520more%2520information,%2520see%2520our%2520copyright%2520guidelines:%2520http:%2Fjournals.plos.org%2Fplosone%2Fs%2Flicenses-and-copyright.%250b%250bWe%2520require%2520you%2520to%2520either%2520(1)%2520present%2520written%2520permission%2520from%2520the%2520copyright%2520holder%2520to%2520publish%2520these%2520figures%2520specifically%2520under%2520the%2520CC%2520BY%25204.0%2520license,%2520or%2520(2)%2520remove%2520the%2520figures%2520from%2520your%2520submission:%250b%250ba.%2520You%2520may%2520seek%2520permission%2520from%2520the%2520original%2520copyright%2520holder%2520of%2520Figure%25204%2520to%2520publish%2520the%2520content%2520specifically%2520under%2520the%2520CC%2520BY%25204.0%2520license.%2520%250b%250bWe%2520recommend%2520that%2520you%2520contact%2520the%2520original%2520copyright%2520holder%2520with%2520the%2520Content%2520Permission%2520Form%2520(http:%2Fjournals.plos.org%2Fplosone%2Fs%2Ffile%3Fid=7c09%2Fcontent-permission-form.pdf)%2520and%2520the%2520following%2520text:%250b%E2%80%9CI%2520request%2520permission%2520for%2520the%2520open-access%2520journal%2520PLOS%2520ONE%2520to%2520publish%2520XXX%2520under%2520the%2520Creative%2520Commons%2520Attribution%2520License%2520(CCAL)%2520CC%2520BY%25204.0%2520(http:%2F%2Fcreativecommons.org%2Flicenses%2Fby%2F4.0%2F).%2520Please%2520be%2520aware%2520that%2520this%2520license%2520allows%2520unrestricted%2520use%2520and%2520distribution,%2520even%2520commercially,%2520by%2520third%2520parties.%2520Please%2520reply%2520and%2520provide%2520explicit%2520written%2520permission%2520to%2520publish%2520XXX%2520under%2520a%2520CC%2520BY%2520license%2520and%2520complete%2520the%2520attached%2520form.%E2%80%9D%250b%250bPlease%2520upload%2520the%2520completed%2520Content%2520Permission%2520Form%2520or%2520other%2520proof%2520of%2520granted%2520permissions%2520as%2520an%2520%2522Other%2522%2520file%2520with%2520your%2520submission.%250b%250bIn%2520the%2520figure%2520caption%2520of%2520the%2520copyrighted%2520figure,%2520please%2520include%2520the%2520following%2520text:%2520%E2%80%9CReprinted%2520from%2520%255bref%255d%2520under%2520a%2520CC%2520BY%2520license,%2520with%2520permission%2520from%2520%255bname%2520of%2520publisher%255d,%2520original%2520copyright%2520%255boriginal%2520copyright%2520year%255d.%E2%80%9D%250b%250bb.%2520If%2520you%2520are%2520unable%2520to%2520obtain%2520permiss/1/010f019e29865dbe-902774bc-9d7d-4da2-8062-6068bc94f825-000000/PNnKSUZsy__Zcl4IdOUTkaqPrlHmA14iOezaA2RB794=258)

[a. You may seek permission from the original copyright holder of Figure 4 to publish the content specifically under the CC BY 4.0 license.](http://track.editorialmanager.com/CL0/We%2520note%2520that%2520Figure%25204%2520in%2520your%2520submission%2520contain%2520%255bmap%2Fsatellite%255d%2520images%2520which%2520may%2520be%2520copyrighted.%2520All%2520PLOS%2520content%2520is%2520published%2520under%2520the%2520Creative%2520Commons%2520Attribution%2520License%2520(CC%2520BY%25204.0),%2520which%2520means%2520that%2520the%2520manuscript,%2520images,%2520and%2520Supporting%2520Information%2520files%2520will%2520be%2520freely%2520available%2520online,%2520and%2520any%2520third%2520party%2520is%2520permitted%2520to%2520access,%2520download,%2520copy,%2520distribute,%2520and%2520use%2520these%2520materials%2520in%2520any%2520way,%2520even%2520commercially,%2520with%2520proper%2520attribution.%2520For%2520these%2520reasons,%2520we%2520cannot%2520publish%2520previously%2520copyrighted%2520maps%2520or%2520satellite%2520images%2520created%2520using%2520proprietary%2520data,%2520such%2520as%2520Google%2520software%2520(Google%2520Maps,%2520Street%2520View,%2520and%2520Earth).%2520For%2520more%2520information,%2520see%2520our%2520copyright%2520guidelines:%2520http:%2Fjournals.plos.org%2Fplosone%2Fs%2Flicenses-and-copyright.%250b%250bWe%2520require%2520you%2520to%2520either%2520(1)%2520present%2520written%2520permission%2520from%2520the%2520copyright%2520holder%2520to%2520publish%2520these%2520figures%2520specifically%2520under%2520the%2520CC%2520BY%25204.0%2520license,%2520or%2520(2)%2520remove%2520the%2520figures%2520from%2520your%2520submission:%250b%250ba.%2520You%2520may%2520seek%2520permission%2520from%2520the%2520original%2520copyright%2520holder%2520of%2520Figure%25204%2520to%2520publish%2520the%2520content%2520specifically%2520under%2520the%2520CC%2520BY%25204.0%2520license.%2520%250b%250bWe%2520recommend%2520that%2520you%2520contact%2520the%2520original%2520copyright%2520holder%2520with%2520the%2520Content%2520Permission%2520Form%2520(http:%2Fjournals.plos.org%2Fplosone%2Fs%2Ffile%3Fid=7c09%2Fcontent-permission-form.pdf)%2520and%2520the%2520following%2520text:%250b%E2%80%9CI%2520request%2520permission%2520for%2520the%2520open-access%2520journal%2520PLOS%2520ONE%2520to%2520publish%2520XXX%2520under%2520the%2520Creative%2520Commons%2520Attribution%2520License%2520(CCAL)%2520CC%2520BY%25204.0%2520(http:%2F%2Fcreativecommons.org%2Flicenses%2Fby%2F4.0%2F).%2520Please%2520be%2520aware%2520that%2520this%2520license%2520allows%2520unrestricted%2520use%2520and%2520distribution,%2520even%2520commercially,%2520by%2520third%2520parties.%2520Please%2520reply%2520and%2520provide%2520explicit%2520written%2520permission%2520to%2520publish%2520XXX%2520under%2520a%2520CC%2520BY%2520license%2520and%2520complete%2520the%2520attached%2520form.%E2%80%9D%250b%250bPlease%2520upload%2520the%2520completed%2520Content%2520Permission%2520Form%2520or%2520other%2520proof%2520of%2520granted%2520permissions%2520as%2520an%2520%2522Other%2522%2520file%2520with%2520your%2520submission.%250b%250bIn%2520the%2520figure%2520caption%2520of%2520the%2520copyrighted%2520figure,%2520please%2520include%2520the%2520following%2520text:%2520%E2%80%9CReprinted%2520from%2520%255bref%255d%2520under%2520a%2520CC%2520BY%2520license,%2520with%2520permission%2520from%2520%255bname%2520of%2520publisher%255d,%2520original%2520copyright%2520%255boriginal%2520copyright%2520year%255d.%E2%80%9D%250b%250bb.%2520If%2520you%2520are%2520unable%2520to%2520obtain%2520permiss/1/010f019e29865dbe-902774bc-9d7d-4da2-8062-6068bc94f825-000000/PNnKSUZsy__Zcl4IdOUTkaqPrlHmA14iOezaA2RB794=258)

[We recommend that you contact the original copyright holder with the Content Permission Form (http://journals.plos.org/plosone/s/file?id=7c09/content-permission-form.pdf) and the following text:](http://track.editorialmanager.com/CL0/We%2520note%2520that%2520Figure%25204%2520in%2520your%2520submission%2520contain%2520%255bmap%2Fsatellite%255d%2520images%2520which%2520may%2520be%2520copyrighted.%2520All%2520PLOS%2520content%2520is%2520published%2520under%2520the%2520Creative%2520Commons%2520Attribution%2520License%2520(CC%2520BY%25204.0),%2520which%2520means%2520that%2520the%2520manuscript,%2520images,%2520and%2520Supporting%2520Information%2520files%2520will%2520be%2520freely%2520available%2520online,%2520and%2520any%2520third%2520party%2520is%2520permitted%2520to%2520access,%2520download,%2520copy,%2520distribute,%2520and%2520use%2520these%2520materials%2520in%2520any%2520way,%2520even%2520commercially,%2520with%2520proper%2520attribution.%2520For%2520these%2520reasons,%2520we%2520cannot%2520publish%2520previously%2520copyrighted%2520maps%2520or%2520satellite%2520images%2520created%2520using%2520proprietary%2520data,%2520such%2520as%2520Google%2520software%2520(Google%2520Maps,%2520Street%2520View,%2520and%2520Earth).%2520For%2520more%2520information,%2520see%2520our%2520copyright%2520guidelines:%2520http:%2Fjournals.plos.org%2Fplosone%2Fs%2Flicenses-and-copyright.%250b%250bWe%2520require%2520you%2520to%2520either%2520(1)%2520present%2520written%2520permission%2520from%2520the%2520copyright%2520holder%2520to%2520publish%2520these%2520figures%2520specifically%2520under%2520the%2520CC%2520BY%25204.0%2520license,%2520or%2520(2)%2520remove%2520the%2520figures%2520from%2520your%2520submission:%250b%250ba.%2520You%2520may%2520seek%2520permission%2520from%2520the%2520original%2520copyright%2520holder%2520of%2520Figure%25204%2520to%2520publish%2520the%2520content%2520specifically%2520under%2520the%2520CC%2520BY%25204.0%2520license.%2520%250b%250bWe%2520recommend%2520that%2520you%2520contact%2520the%2520original%2520copyright%2520holder%2520with%2520the%2520Content%2520Permission%2520Form%2520(http:%2Fjournals.plos.org%2Fplosone%2Fs%2Ffile%3Fid=7c09%2Fcontent-permission-form.pdf)%2520and%2520the%2520following%2520text:%250b%E2%80%9CI%2520request%2520permission%2520for%2520the%2520open-access%2520journal%2520PLOS%2520ONE%2520to%2520publish%2520XXX%2520under%2520the%2520Creative%2520Commons%2520Attribution%2520License%2520(CCAL)%2520CC%2520BY%25204.0%2520(http:%2F%2Fcreativecommons.org%2Flicenses%2Fby%2F4.0%2F).%2520Please%2520be%2520aware%2520that%2520this%2520license%2520allows%2520unrestricted%2520use%2520and%2520distribution,%2520even%2520commercially,%2520by%2520third%2520parties.%2520Please%2520reply%2520and%2520provide%2520explicit%2520written%2520permission%2520to%2520publish%2520XXX%2520under%2520a%2520CC%2520BY%2520license%2520and%2520complete%2520the%2520attached%2520form.%E2%80%9D%250b%250bPlease%2520upload%2520the%2520completed%2520Content%2520Permission%2520Form%2520or%2520other%2520proof%2520of%2520granted%2520permissions%2520as%2520an%2520%2522Other%2522%2520file%2520with%2520your%2520submission.%250b%250bIn%2520the%2520figure%2520caption%2520of%2520the%2520copyrighted%2520figure,%2520please%2520include%2520the%2520following%2520text:%2520%E2%80%9CReprinted%2520from%2520%255bref%255d%2520under%2520a%2520CC%2520BY%2520license,%2520with%2520permission%2520from%2520%255bname%2520of%2520publisher%255d,%2520original%2520copyright%2520%255boriginal%2520copyright%2520year%255d.%E2%80%9D%250b%250bb.%2520If%2520you%2520are%2520unable%2520to%2520obtain%2520permiss/1/010f019e29865dbe-902774bc-9d7d-4da2-8062-6068bc94f825-000000/PNnKSUZsy__Zcl4IdOUTkaqPrlHmA14iOezaA2RB794=258)

[“I request permission for the open-access journal PLOS ONE to publish XXX under the Creative Commons Attribution License (CCAL) CC BY 4.0 (http://creativecommons.org/licenses/by/4.0/). Please be aware that this license allows unrestricted use and distribution, even commercially, by third parties. Please reply and provide explicit written permission to publish XXX under a CC BY license and complete the attached form.”](http://track.editorialmanager.com/CL0/We%2520note%2520that%2520Figure%25204%2520in%2520your%2520submission%2520contain%2520%255bmap%2Fsatellite%255d%2520images%2520which%2520may%2520be%2520copyrighted.%2520All%2520PLOS%2520content%2520is%2520published%2520under%2520the%2520Creative%2520Commons%2520Attribution%2520License%2520(CC%2520BY%25204.0),%2520which%2520means%2520that%2520the%2520manuscript,%2520images,%2520and%2520Supporting%2520Information%2520files%2520will%2520be%2520freely%2520available%2520online,%2520and%2520any%2520third%2520party%2520is%2520permitted%2520to%2520access,%2520download,%2520copy,%2520distribute,%2520and%2520use%2520these%2520materials%2520in%2520any%2520way,%2520even%2520commercially,%2520with%2520proper%2520attribution.%2520For%2520these%2520reasons,%2520we%2520cannot%2520publish%2520previously%2520copyrighted%2520maps%2520or%2520satellite%2520images%2520created%2520using%2520proprietary%2520data,%2520such%2520as%2520Google%2520software%2520(Google%2520Maps,%2520Street%2520View,%2520and%2520Earth).%2520For%2520more%2520information,%2520see%2520our%2520copyright%2520guidelines:%2520http:%2Fjournals.plos.org%2Fplosone%2Fs%2Flicenses-and-copyright.%250b%250bWe%2520require%2520you%2520to%2520either%2520(1)%2520present%2520written%2520permission%2520from%2520the%2520copyright%2520holder%2520to%2520publish%2520these%2520figures%2520specifically%2520under%2520the%2520CC%2520BY%25204.0%2520license,%2520or%2520(2)%2520remove%2520the%2520figures%2520from%2520your%2520submission:%250b%250ba.%2520You%2520may%2520seek%2520permission%2520from%2520the%2520original%2520copyright%2520holder%2520of%2520Figure%25204%2520to%2520publish%2520the%2520content%2520specifically%2520under%2520the%2520CC%2520BY%25204.0%2520license.%2520%250b%250bWe%2520recommend%2520that%2520you%2520contact%2520the%2520original%2520copyright%2520holder%2520with%2520the%2520Content%2520Permission%2520Form%2520(http:%2Fjournals.plos.org%2Fplosone%2Fs%2Ffile%3Fid=7c09%2Fcontent-permission-form.pdf)%2520and%2520the%2520following%2520text:%250b%E2%80%9CI%2520request%2520permission%2520for%2520the%2520open-access%2520journal%2520PLOS%2520ONE%2520to%2520publish%2520XXX%2520under%2520the%2520Creative%2520Commons%2520Attribution%2520License%2520(CCAL)%2520CC%2520BY%25204.0%2520(http:%2F%2Fcreativecommons.org%2Flicenses%2Fby%2F4.0%2F).%2520Please%2520be%2520aware%2520that%2520this%2520license%2520allows%2520unrestricted%2520use%2520and%2520distribution,%2520even%2520commercially,%2520by%2520third%2520parties.%2520Please%2520reply%2520and%2520provide%2520explicit%2520written%2520permission%2520to%2520publish%2520XXX%2520under%2520a%2520CC%2520BY%2520license%2520and%2520complete%2520the%2520attached%2520form.%E2%80%9D%250b%250bPlease%2520upload%2520the%2520completed%2520Content%2520Permission%2520Form%2520or%2520other%2520proof%2520of%2520granted%2520permissions%2520as%2520an%2520%2522Other%2522%2520file%2520with%2520your%2520submission.%250b%250bIn%2520the%2520figure%2520caption%2520of%2520the%2520copyrighted%2520figure,%2520please%2520include%2520the%2520following%2520text:%2520%E2%80%9CReprinted%2520from%2520%255bref%255d%2520under%2520a%2520CC%2520BY%2520license,%2520with%2520permission%2520from%2520%255bname%2520of%2520publisher%255d,%2520original%2520copyright%2520%255boriginal%2520copyright%2520year%255d.%E2%80%9D%250b%250bb.%2520If%2520you%2520are%2520unable%2520to%2520obtain%2520permiss/1/010f019e29865dbe-902774bc-9d7d-4da2-8062-6068bc94f825-000000/PNnKSUZsy__Zcl4IdOUTkaqPrlHmA14iOezaA2RB794=258)

[Please upload the completed Content Permission Form or other proof of granted permissions as an "Other" file with your submission.](http://track.editorialmanager.com/CL0/We%2520note%2520that%2520Figure%25204%2520in%2520your%2520submission%2520contain%2520%255bmap%2Fsatellite%255d%2520images%2520which%2520may%2520be%2520copyrighted.%2520All%2520PLOS%2520content%2520is%2520published%2520under%2520the%2520Creative%2520Commons%2520Attribution%2520License%2520(CC%2520BY%25204.0),%2520which%2520means%2520that%2520the%2520manuscript,%2520images,%2520and%2520Supporting%2520Information%2520files%2520will%2520be%2520freely%2520available%2520online,%2520and%2520any%2520third%2520party%2520is%2520permitted%2520to%2520access,%2520download,%2520copy,%2520distribute,%2520and%2520use%2520these%2520materials%2520in%2520any%2520way,%2520even%2520commercially,%2520with%2520proper%2520attribution.%2520For%2520these%2520reasons,%2520we%2520cannot%2520publish%2520previously%2520copyrighted%2520maps%2520or%2520satellite%2520images%2520created%2520using%2520proprietary%2520data,%2520such%2520as%2520Google%2520software%2520(Google%2520Maps,%2520Street%2520View,%2520and%2520Earth).%2520For%2520more%2520information,%2520see%2520our%2520copyright%2520guidelines:%2520http:%2Fjournals.plos.org%2Fplosone%2Fs%2Flicenses-and-copyright.%250b%250bWe%2520require%2520you%2520to%2520either%2520(1)%2520present%2520written%2520permission%2520from%2520the%2520copyright%2520holder%2520to%2520publish%2520these%2520figures%2520specifically%2520under%2520the%2520CC%2520BY%25204.0%2520license,%2520or%2520(2)%2520remove%2520the%2520figures%2520from%2520your%2520submission:%250b%250ba.%2520You%2520may%2520seek%2520permission%2520from%2520the%2520original%2520copyright%2520holder%2520of%2520Figure%25204%2520to%2520publish%2520the%2520content%2520specifically%2520under%2520the%2520CC%2520BY%25204.0%2520license.%2520%250b%250bWe%2520recommend%2520that%2520you%2520contact%2520the%2520original%2520copyright%2520holder%2520with%2520the%2520Content%2520Permission%2520Form%2520(http:%2Fjournals.plos.org%2Fplosone%2Fs%2Ffile%3Fid=7c09%2Fcontent-permission-form.pdf)%2520and%2520the%2520following%2520text:%250b%E2%80%9CI%2520request%2520permission%2520for%2520the%2520open-access%2520journal%2520PLOS%2520ONE%2520to%2520publish%2520XXX%2520under%2520the%2520Creative%2520Commons%2520Attribution%2520License%2520(CCAL)%2520CC%2520BY%25204.0%2520(http:%2F%2Fcreativecommons.org%2Flicenses%2Fby%2F4.0%2F).%2520Please%2520be%2520aware%2520that%2520this%2520license%2520allows%2520unrestricted%2520use%2520and%2520distribution,%2520even%2520commercially,%2520by%2520third%2520parties.%2520Please%2520reply%2520and%2520provide%2520explicit%2520written%2520permission%2520to%2520publish%2520XXX%2520under%2520a%2520CC%2520BY%2520license%2520and%2520complete%2520the%2520attached%2520form.%E2%80%9D%250b%250bPlease%2520upload%2520the%2520completed%2520Content%2520Permission%2520Form%2520or%2520other%2520proof%2520of%2520granted%2520permissions%2520as%2520an%2520%2522Other%2522%2520file%2520with%2520your%2520submission.%250b%250bIn%2520the%2520figure%2520caption%2520of%2520the%2520copyrighted%2520figure,%2520please%2520include%2520the%2520following%2520text:%2520%E2%80%9CReprinted%2520from%2520%255bref%255d%2520under%2520a%2520CC%2520BY%2520license,%2520with%2520permission%2520from%2520%255bname%2520of%2520publisher%255d,%2520original%2520copyright%2520%255boriginal%2520copyright%2520year%255d.%E2%80%9D%250b%250bb.%2520If%2520you%2520are%2520unable%2520to%2520obtain%2520permiss/1/010f019e29865dbe-902774bc-9d7d-4da2-8062-6068bc94f825-000000/PNnKSUZsy__Zcl4IdOUTkaqPrlHmA14iOezaA2RB794=258)

[In the figure caption of the copyrighted figure, please include the following text: “Reprinted from [ref] under a CC BY license, with permission from [name of publisher], original copyright [original copyright year].”](http://track.editorialmanager.com/CL0/We%2520note%2520that%2520Figure%25204%2520in%2520your%2520submission%2520contain%2520%255bmap%2Fsatellite%255d%2520images%2520which%2520may%2520be%2520copyrighted.%2520All%2520PLOS%2520content%2520is%2520published%2520under%2520the%2520Creative%2520Commons%2520Attribution%2520License%2520(CC%2520BY%25204.0),%2520which%2520means%2520that%2520the%2520manuscript,%2520images,%2520and%2520Supporting%2520Information%2520files%2520will%2520be%2520freely%2520available%2520online,%2520and%2520any%2520third%2520party%2520is%2520permitted%2520to%2520access,%2520download,%2520copy,%2520distribute,%2520and%2520use%2520these%2520materials%2520in%2520any%2520way,%2520even%2520commercially,%2520with%2520proper%2520attribution.%2520For%2520these%2520reasons,%2520we%2520cannot%2520publish%2520previously%2520copyrighted%2520maps%2520or%2520satellite%2520images%2520created%2520using%2520proprietary%2520data,%2520such%2520as%2520Google%2520software%2520(Google%2520Maps,%2520Street%2520View,%2520and%2520Earth).%2520For%2520more%2520information,%2520see%2520our%2520copyright%2520guidelines:%2520http:%2Fjournals.plos.org%2Fplosone%2Fs%2Flicenses-and-copyright.%250b%250bWe%2520require%2520you%2520to%2520either%2520(1)%2520present%2520written%2520permission%2520from%2520the%2520copyright%2520holder%2520to%2520publish%2520these%2520figures%2520specifically%2520under%2520the%2520CC%2520BY%25204.0%2520license,%2520or%2520(2)%2520remove%2520the%2520figures%2520from%2520your%2520submission:%250b%250ba.%2520You%2520may%2520seek%2520permission%2520from%2520the%2520original%2520copyright%2520holder%2520of%2520Figure%25204%2520to%2520publish%2520the%2520content%2520specifically%2520under%2520the%2520CC%2520BY%25204.0%2520license.%2520%250b%250bWe%2520recommend%2520that%2520you%2520contact%2520the%2520original%2520copyright%2520holder%2520with%2520the%2520Content%2520Permission%2520Form%2520(http:%2Fjournals.plos.org%2Fplosone%2Fs%2Ffile%3Fid=7c09%2Fcontent-permission-form.pdf)%2520and%2520the%2520following%2520text:%250b%E2%80%9CI%2520request%2520permission%2520for%2520the%2520open-access%2520journal%2520PLOS%2520ONE%2520to%2520publish%2520XXX%2520under%2520the%2520Creative%2520Commons%2520Attribution%2520License%2520(CCAL)%2520CC%2520BY%25204.0%2520(http:%2F%2Fcreativecommons.org%2Flicenses%2Fby%2F4.0%2F).%2520Please%2520be%2520aware%2520that%2520this%2520license%2520allows%2520unrestricted%2520use%2520and%2520distribution,%2520even%2520commercially,%2520by%2520third%2520parties.%2520Please%2520reply%2520and%2520provide%2520explicit%2520written%2520permission%2520to%2520publish%2520XXX%2520under%2520a%2520CC%2520BY%2520license%2520and%2520complete%2520the%2520attached%2520form.%E2%80%9D%250b%250bPlease%2520upload%2520the%2520completed%2520Content%2520Permission%2520Form%2520or%2520other%2520proof%2520of%2520granted%2520permissions%2520as%2520an%2520%2522Other%2522%2520file%2520with%2520your%2520submission.%250b%250bIn%2520the%2520figure%2520caption%2520of%2520the%2520copyrighted%2520figure,%2520please%2520include%2520the%2520following%2520text:%2520%E2%80%9CReprinted%2520from%2520%255bref%255d%2520under%2520a%2520CC%2520BY%2520license,%2520with%2520permission%2520from%2520%255bname%2520of%2520publisher%255d,%2520original%2520copyright%2520%255boriginal%2520copyright%2520year%255d.%E2%80%9D%250b%250bb.%2520If%2520you%2520are%2520unable%2520to%2520obtain%2520permiss/1/010f019e29865dbe-902774bc-9d7d-4da2-8062-6068bc94f825-000000/PNnKSUZsy__Zcl4IdOUTkaqPrlHmA14iOezaA2RB794=258)

[b. If you are unable to obtain permission from the original copyright holder to publish these figures under the CC BY 4.0 license or if the copyright holder’s requirements are incompatible with the CC BY 4.0 license, please either i) remove the figure or ii) supply a replacement figure that complies with the CC BY 4.0 license. Please check copyright information on all replacement figures and update the figure caption with source information. If applicable, please specify in the figure caption text when a figure is similar but not identical to the original image and is therefore for illustrative purposes only.](http://track.editorialmanager.com/CL0/We%2520note%2520that%2520Figure%25204%2520in%2520your%2520submission%2520contain%2520%255bmap%2Fsatellite%255d%2520images%2520which%2520may%2520be%2520copyrighted.%2520All%2520PLOS%2520content%2520is%2520published%2520under%2520the%2520Creative%2520Commons%2520Attribution%2520License%2520(CC%2520BY%25204.0),%2520which%2520means%2520that%2520the%2520manuscript,%2520images,%2520and%2520Supporting%2520Information%2520files%2520will%2520be%2520freely%2520available%2520online,%2520and%2520any%2520third%2520party%2520is%2520permitted%2520to%2520access,%2520download,%2520copy,%2520distribute,%2520and%2520use%2520these%2520materials%2520in%2520any%2520way,%2520even%2520commercially,%2520with%2520proper%2520attribution.%2520For%2520these%2520reasons,%2520we%2520cannot%2520publish%2520previously%2520copyrighted%2520maps%2520or%2520satellite%2520images%2520created%2520using%2520proprietary%2520data,%2520such%2520as%2520Google%2520software%2520(Google%2520Maps,%2520Street%2520View,%2520and%2520Earth).%2520For%2520more%2520information,%2520see%2520our%2520copyright%2520guidelines:%2520http:%2Fjournals.plos.org%2Fplosone%2Fs%2Flicenses-and-copyright.%250b%250bWe%2520require%2520you%2520to%2520either%2520(1)%2520present%2520written%2520permission%2520from%2520the%2520copyright%2520holder%2520to%2520publish%2520these%2520figures%2520specifically%2520under%2520the%2520CC%2520BY%25204.0%2520license,%2520or%2520(2)%2520remove%2520the%2520figures%2520from%2520your%2520submission:%250b%250ba.%2520You%2520may%2520seek%2520permission%2520from%2520the%2520original%2520copyright%2520holder%2520of%2520Figure%25204%2520to%2520publish%2520the%2520content%2520specifically%2520under%2520the%2520CC%2520BY%25204.0%2520license.%2520%250b%250bWe%2520recommend%2520that%2520you%2520contact%2520the%2520original%2520copyright%2520holder%2520with%2520the%2520Content%2520Permission%2520Form%2520(http:%2Fjournals.plos.org%2Fplosone%2Fs%2Ffile%3Fid=7c09%2Fcontent-permission-form.pdf)%2520and%2520the%2520following%2520text:%250b%E2%80%9CI%2520request%2520permission%2520for%2520the%2520open-access%2520journal%2520PLOS%2520ONE%2520to%2520publish%2520XXX%2520under%2520the%2520Creative%2520Commons%2520Attribution%2520License%2520(CCAL)%2520CC%2520BY%25204.0%2520(http:%2F%2Fcreativecommons.org%2Flicenses%2Fby%2F4.0%2F).%2520Please%2520be%2520aware%2520that%2520this%2520license%2520allows%2520unrestricted%2520use%2520and%2520distribution,%2520even%2520commercially,%2520by%2520third%2520parties.%2520Please%2520reply%2520and%2520provide%2520explicit%2520written%2520permission%2520to%2520publish%2520XXX%2520under%2520a%2520CC%2520BY%2520license%2520and%2520complete%2520the%2520attached%2520form.%E2%80%9D%250b%250bPlease%2520upload%2520the%2520completed%2520Content%2520Permission%2520Form%2520or%2520other%2520proof%2520of%2520granted%2520permissions%2520as%2520an%2520%2522Other%2522%2520file%2520with%2520your%2520submission.%250b%250bIn%2520the%2520figure%2520caption%2520of%2520the%2520copyrighted%2520figure,%2520please%2520include%2520the%2520following%2520text:%2520%E2%80%9CReprinted%2520from%2520%255bref%255d%2520under%2520a%2520CC%2520BY%2520license,%2520with%2520permission%2520from%2520%255bname%2520of%2520publisher%255d,%2520original%2520copyright%2520%255boriginal%2520copyright%2520year%255d.%E2%80%9D%250b%250bb.%2520If%2520you%2520are%2520unable%2520to%2520obtain%2520permiss/1/010f019e29865dbe-902774bc-9d7d-4da2-8062-6068bc94f825-000000/PNnKSUZsy__Zcl4IdOUTkaqPrlHmA14iOezaA2RB794=258)

[The following resources for replacing copyrighted map figures may be helpful:](http://track.editorialmanager.com/CL0/We%2520note%2520that%2520Figure%25204%2520in%2520your%2520submission%2520contain%2520%255bmap%2Fsatellite%255d%2520images%2520which%2520may%2520be%2520copyrighted.%2520All%2520PLOS%2520content%2520is%2520published%2520under%2520the%2520Creative%2520Commons%2520Attribution%2520License%2520(CC%2520BY%25204.0),%2520which%2520means%2520that%2520the%2520manuscript,%2520images,%2520and%2520Supporting%2520Information%2520files%2520will%2520be%2520freely%2520available%2520online,%2520and%2520any%2520third%2520party%2520is%2520permitted%2520to%2520access,%2520download,%2520copy,%2520distribute,%2520and%2520use%2520these%2520materials%2520in%2520any%2520way,%2520even%2520commercially,%2520with%2520proper%2520attribution.%2520For%2520these%2520reasons,%2520we%2520cannot%2520publish%2520previously%2520copyrighted%2520maps%2520or%2520satellite%2520images%2520created%2520using%2520proprietary%2520data,%2520such%2520as%2520Google%2520software%2520(Google%2520Maps,%2520Street%2520View,%2520and%2520Earth).%2520For%2520more%2520information,%2520see%2520our%2520copyright%2520guidelines:%2520http:%2Fjournals.plos.org%2Fplosone%2Fs%2Flicenses-and-copyright.%250b%250bWe%2520require%2520you%2520to%2520either%2520(1)%2520present%2520written%2520permission%2520from%2520the%2520copyright%2520holder%2520to%2520publish%2520these%2520figures%2520specifically%2520under%2520the%2520CC%2520BY%25204.0%2520license,%2520or%2520(2)%2520remove%2520the%2520figures%2520from%2520your%2520submission:%250b%250ba.%2520You%2520may%2520seek%2520permission%2520from%2520the%2520original%2520copyright%2520holder%2520of%2520Figure%25204%2520to%2520publish%2520the%2520content%2520specifically%2520under%2520the%2520CC%2520BY%25204.0%2520license.%2520%250b%250bWe%2520recommend%2520that%2520you%2520contact%2520the%2520original%2520copyright%2520holder%2520with%2520the%2520Content%2520Permission%2520Form%2520(http:%2Fjournals.plos.org%2Fplosone%2Fs%2Ffile%3Fid=7c09%2Fcontent-permission-form.pdf)%2520and%2520the%2520following%2520text:%250b%E2%80%9CI%2520request%2520permission%2520for%2520the%2520open-access%2520journal%2520PLOS%2520ONE%2520to%2520publish%2520XXX%2520under%2520the%2520Creative%2520Commons%2520Attribution%2520License%2520(CCAL)%2520CC%2520BY%25204.0%2520(http:%2F%2Fcreativecommons.org%2Flicenses%2Fby%2F4.0%2F).%2520Please%2520be%2520aware%2520that%2520this%2520license%2520allows%2520unrestricted%2520use%2520and%2520distribution,%2520even%2520commercially,%2520by%2520third%2520parties.%2520Please%2520reply%2520and%2520provide%2520explicit%2520written%2520permission%2520to%2520publish%2520XXX%2520under%2520a%2520CC%2520BY%2520license%2520and%2520complete%2520the%2520attached%2520form.%E2%80%9D%250b%250bPlease%2520upload%2520the%2520completed%2520Content%2520Permission%2520Form%2520or%2520other%2520proof%2520of%2520granted%2520permissions%2520as%2520an%2520%2522Other%2522%2520file%2520with%2520your%2520submission.%250b%250bIn%2520the%2520figure%2520caption%2520of%2520the%2520copyrighted%2520figure,%2520please%2520include%2520the%2520following%2520text:%2520%E2%80%9CReprinted%2520from%2520%255bref%255d%2520under%2520a%2520CC%2520BY%2520license,%2520with%2520permission%2520from%2520%255bname%2520of%2520publisher%255d,%2520original%2520copyright%2520%255boriginal%2520copyright%2520year%255d.%E2%80%9D%250b%250bb.%2520If%2520you%2520are%2520unable%2520to%2520obtain%2520permiss/1/010f019e29865dbe-902774bc-9d7d-4da2-8062-6068bc94f825-000000/PNnKSUZsy__Zcl4IdOUTkaqPrlHmA14iOezaA2RB794=258)

[USGS National Map Viewer (public domain): http://viewer.nationalmap.gov/viewer/](http://track.editorialmanager.com/CL0/We%2520note%2520that%2520Figure%25204%2520in%2520your%2520submission%2520contain%2520%255bmap%2Fsatellite%255d%2520images%2520which%2520may%2520be%2520copyrighted.%2520All%2520PLOS%2520content%2520is%2520published%2520under%2520the%2520Creative%2520Commons%2520Attribution%2520License%2520(CC%2520BY%25204.0),%2520which%2520means%2520that%2520the%2520manuscript,%2520images,%2520and%2520Supporting%2520Information%2520files%2520will%2520be%2520freely%2520available%2520online,%2520and%2520any%2520third%2520party%2520is%2520permitted%2520to%2520access,%2520download,%2520copy,%2520distribute,%2520and%2520use%2520these%2520materials%2520in%2520any%2520way,%2520even%2520commercially,%2520with%2520proper%2520attribution.%2520For%2520these%2520reasons,%2520we%2520cannot%2520publish%2520previously%2520copyrighted%2520maps%2520or%2520satellite%2520images%2520created%2520using%2520proprietary%2520data,%2520such%2520as%2520Google%2520software%2520(Google%2520Maps,%2520Street%2520View,%2520and%2520Earth).%2520For%2520more%2520information,%2520see%2520our%2520copyright%2520guidelines:%2520http:%2Fjournals.plos.org%2Fplosone%2Fs%2Flicenses-and-copyright.%250b%250bWe%2520require%2520you%2520to%2520either%2520(1)%2520present%2520written%2520permission%2520from%2520the%2520copyright%2520holder%2520to%2520publish%2520these%2520figures%2520specifically%2520under%2520the%2520CC%2520BY%25204.0%2520license,%2520or%2520(2)%2520remove%2520the%2520figures%2520from%2520your%2520submission:%250b%250ba.%2520You%2520may%2520seek%2520permission%2520from%2520the%2520original%2520copyright%2520holder%2520of%2520Figure%25204%2520to%2520publish%2520the%2520content%2520specifically%2520under%2520the%2520CC%2520BY%25204.0%2520license.%2520%250b%250bWe%2520recommend%2520that%2520you%2520contact%2520the%2520original%2520copyright%2520holder%2520with%2520the%2520Content%2520Permission%2520Form%2520(http:%2Fjournals.plos.org%2Fplosone%2Fs%2Ffile%3Fid=7c09%2Fcontent-permission-form.pdf)%2520and%2520the%2520following%2520text:%250b%E2%80%9CI%2520request%2520permission%2520for%2520the%2520open-access%2520journal%2520PLOS%2520ONE%2520to%2520publish%2520XXX%2520under%2520the%2520Creative%2520Commons%2520Attribution%2520License%2520(CCAL)%2520CC%2520BY%25204.0%2520(http:%2F%2Fcreativecommons.org%2Flicenses%2Fby%2F4.0%2F).%2520Please%2520be%2520aware%2520that%2520this%2520license%2520allows%2520unrestricted%2520use%2520and%2520distribution,%2520even%2520commercially,%2520by%2520third%2520parties.%2520Please%2520reply%2520and%2520provide%2520explicit%2520written%2520permission%2520to%2520publish%2520XXX%2520under%2520a%2520CC%2520BY%2520license%2520and%2520complete%2520the%2520attached%2520form.%E2%80%9D%250b%250bPlease%2520upload%2520the%2520completed%2520Content%2520Permission%2520Form%2520or%2520other%2520proof%2520of%2520granted%2520permissions%2520as%2520an%2520%2522Other%2522%2520file%2520with%2520your%2520submission.%250b%250bIn%2520the%2520figure%2520caption%2520of%2520the%2520copyrighted%2520figure,%2520please%2520include%2520the%2520following%2520text:%2520%E2%80%9CReprinted%2520from%2520%255bref%255d%2520under%2520a%2520CC%2520BY%2520license,%2520with%2520permission%2520from%2520%255bname%2520of%2520publisher%255d,%2520original%2520copyright%2520%255boriginal%2520copyright%2520year%255d.%E2%80%9D%250b%250bb.%2520If%2520you%2520are%2520unable%2520to%2520obtain%2520permiss/1/010f019e29865dbe-902774bc-9d7d-4da2-8062-6068bc94f825-000000/PNnKSUZsy__Zcl4IdOUTkaqPrlHmA14iOezaA2RB794=258)

[The Gateway to Astronaut Photography of Earth (public domain): http://eol.jsc.nasa.gov/sseop/clickmap/](http://track.editorialmanager.com/CL0/We%2520note%2520that%2520Figure%25204%2520in%2520your%2520submission%2520contain%2520%255bmap%2Fsatellite%255d%2520images%2520which%2520may%2520be%2520copyrighted.%2520All%2520PLOS%2520content%2520is%2520published%2520under%2520the%2520Creative%2520Commons%2520Attribution%2520License%2520(CC%2520BY%25204.0),%2520which%2520means%2520that%2520the%2520manuscript,%2520images,%2520and%2520Supporting%2520Information%2520files%2520will%2520be%2520freely%2520available%2520online,%2520and%2520any%2520third%2520party%2520is%2520permitted%2520to%2520access,%2520download,%2520copy,%2520distribute,%2520and%2520use%2520these%2520materials%2520in%2520any%2520way,%2520even%2520commercially,%2520with%2520proper%2520attribution.%2520For%2520these%2520reasons,%2520we%2520cannot%2520publish%2520previously%2520copyrighted%2520maps%2520or%2520satellite%2520images%2520created%2520using%2520proprietary%2520data,%2520such%2520as%2520Google%2520software%2520(Google%2520Maps,%2520Street%2520View,%2520and%2520Earth).%2520For%2520more%2520information,%2520see%2520our%2520copyright%2520guidelines:%2520http:%2Fjournals.plos.org%2Fplosone%2Fs%2Flicenses-and-copyright.%250b%250bWe%2520require%2520you%2520to%2520either%2520(1)%2520present%2520written%2520permission%2520from%2520the%2520copyright%2520holder%2520to%2520publish%2520these%2520figures%2520specifically%2520under%2520the%2520CC%2520BY%25204.0%2520license,%2520or%2520(2)%2520remove%2520the%2520figures%2520from%2520your%2520submission:%250b%250ba.%2520You%2520may%2520seek%2520permission%2520from%2520the%2520original%2520copyright%2520holder%2520of%2520Figure%25204%2520to%2520publish%2520the%2520content%2520specifically%2520under%2520the%2520CC%2520BY%25204.0%2520license.%2520%250b%250bWe%2520recommend%2520that%2520you%2520contact%2520the%2520original%2520copyright%2520holder%2520with%2520the%2520Content%2520Permission%2520Form%2520(http:%2Fjournals.plos.org%2Fplosone%2Fs%2Ffile%3Fid=7c09%2Fcontent-permission-form.pdf)%2520and%2520the%2520following%2520text:%250b%E2%80%9CI%2520request%2520permission%2520for%2520the%2520open-access%2520journal%2520PLOS%2520ONE%2520to%2520publish%2520XXX%2520under%2520the%2520Creative%2520Commons%2520Attribution%2520License%2520(CCAL)%2520CC%2520BY%25204.0%2520(http:%2F%2Fcreativecommons.org%2Flicenses%2Fby%2F4.0%2F).%2520Please%2520be%2520aware%2520that%2520this%2520license%2520allows%2520unrestricted%2520use%2520and%2520distribution,%2520even%2520commercially,%2520by%2520third%2520parties.%2520Please%2520reply%2520and%2520provide%2520explicit%2520written%2520permission%2520to%2520publish%2520XXX%2520under%2520a%2520CC%2520BY%2520license%2520and%2520complete%2520the%2520attached%2520form.%E2%80%9D%250b%250bPlease%2520upload%2520the%2520completed%2520Content%2520Permission%2520Form%2520or%2520other%2520proof%2520of%2520granted%2520permissions%2520as%2520an%2520%2522Other%2522%2520file%2520with%2520your%2520submission.%250b%250bIn%2520the%2520figure%2520caption%2520of%2520the%2520copyrighted%2520figure,%2520please%2520include%2520the%2520following%2520text:%2520%E2%80%9CReprinted%2520from%2520%255bref%255d%2520under%2520a%2520CC%2520BY%2520license,%2520with%2520permission%2520from%2520%255bname%2520of%2520publisher%255d,%2520original%2520copyright%2520%255boriginal%2520copyright%2520year%255d.%E2%80%9D%250b%250bb.%2520If%2520you%2520are%2520unable%2520to%2520obtain%2520permiss/1/010f019e29865dbe-902774bc-9d7d-4da2-8062-6068bc94f825-000000/PNnKSUZsy__Zcl4IdOUTkaqPrlHmA14iOezaA2RB794=258)

[Maps at the CIA (public domain): https://www.cia.gov/library/publications/the-world-factbook/index.html and https://www.cia.gov/library/publications/cia-maps-publications/index.html](http://track.editorialmanager.com/CL0/We%2520note%2520that%2520Figure%25204%2520in%2520your%2520submission%2520contain%2520%255bmap%2Fsatellite%255d%2520images%2520which%2520may%2520be%2520copyrighted.%2520All%2520PLOS%2520content%2520is%2520published%2520under%2520the%2520Creative%2520Commons%2520Attribution%2520License%2520(CC%2520BY%25204.0),%2520which%2520means%2520that%2520the%2520manuscript,%2520images,%2520and%2520Supporting%2520Information%2520files%2520will%2520be%2520freely%2520available%2520online,%2520and%2520any%2520third%2520party%2520is%2520permitted%2520to%2520access,%2520download,%2520copy,%2520distribute,%2520and%2520use%2520these%2520materials%2520in%2520any%2520way,%2520even%2520commercially,%2520with%2520proper%2520attribution.%2520For%2520these%2520reasons,%2520we%2520cannot%2520publish%2520previously%2520copyrighted%2520maps%2520or%2520satellite%2520images%2520created%2520using%2520proprietary%2520data,%2520such%2520as%2520Google%2520software%2520(Google%2520Maps,%2520Street%2520View,%2520and%2520Earth).%2520For%2520more%2520information,%2520see%2520our%2520copyright%2520guidelines:%2520http:%2Fjournals.plos.org%2Fplosone%2Fs%2Flicenses-and-copyright.%250b%250bWe%2520require%2520you%2520to%2520either%2520(1)%2520present%2520written%2520permission%2520from%2520the%2520copyright%2520holder%2520to%2520publish%2520these%2520figures%2520specifically%2520under%2520the%2520CC%2520BY%25204.0%2520license,%2520or%2520(2)%2520remove%2520the%2520figures%2520from%2520your%2520submission:%250b%250ba.%2520You%2520may%2520seek%2520permission%2520from%2520the%2520original%2520copyright%2520holder%2520of%2520Figure%25204%2520to%2520publish%2520the%2520content%2520specifically%2520under%2520the%2520CC%2520BY%25204.0%2520license.%2520%250b%250bWe%2520recommend%2520that%2520you%2520contact%2520the%2520original%2520copyright%2520holder%2520with%2520the%2520Content%2520Permission%2520Form%2520(http:%2Fjournals.plos.org%2Fplosone%2Fs%2Ffile%3Fid=7c09%2Fcontent-permission-form.pdf)%2520and%2520the%2520following%2520text:%250b%E2%80%9CI%2520request%2520permission%2520for%2520the%2520open-access%2520journal%2520PLOS%2520ONE%2520to%2520publish%2520XXX%2520under%2520the%2520Creative%2520Commons%2520Attribution%2520License%2520(CCAL)%2520CC%2520BY%25204.0%2520(http:%2F%2Fcreativecommons.org%2Flicenses%2Fby%2F4.0%2F).%2520Please%2520be%2520aware%2520that%2520this%2520license%2520allows%2520unrestricted%2520use%2520and%2520distribution,%2520even%2520commercially,%2520by%2520third%2520parties.%2520Please%2520reply%2520and%2520provide%2520explicit%2520written%2520permission%2520to%2520publish%2520XXX%2520under%2520a%2520CC%2520BY%2520license%2520and%2520complete%2520the%2520attached%2520form.%E2%80%9D%250b%250bPlease%2520upload%2520the%2520completed%2520Content%2520Permission%2520Form%2520or%2520other%2520proof%2520of%2520granted%2520permissions%2520as%2520an%2520%2522Other%2522%2520file%2520with%2520your%2520submission.%250b%250bIn%2520the%2520figure%2520caption%2520of%2520the%2520copyrighted%2520figure,%2520please%2520include%2520the%2520following%2520text:%2520%E2%80%9CReprinted%2520from%2520%255bref%255d%2520under%2520a%2520CC%2520BY%2520license,%2520with%2520permission%2520from%2520%255bname%2520of%2520publisher%255d,%2520original%2520copyright%2520%255boriginal%2520copyright%2520year%255d.%E2%80%9D%250b%250bb.%2520If%2520you%2520are%2520unable%2520to%2520obtain%2520permiss/1/010f019e29865dbe-902774bc-9d7d-4da2-8062-6068bc94f825-000000/PNnKSUZsy__Zcl4IdOUTkaqPrlHmA14iOezaA2RB794=258)

[NASA Earth Observatory (public domain): http://earthobservatory.nasa.gov/](http://track.editorialmanager.com/CL0/We%2520note%2520that%2520Figure%25204%2520in%2520your%2520submission%2520contain%2520%255bmap%2Fsatellite%255d%2520images%2520which%2520may%2520be%2520copyrighted.%2520All%2520PLOS%2520content%2520is%2520published%2520under%2520the%2520Creative%2520Commons%2520Attribution%2520License%2520(CC%2520BY%25204.0),%2520which%2520means%2520that%2520the%2520manuscript,%2520images,%2520and%2520Supporting%2520Information%2520files%2520will%2520be%2520freely%2520available%2520online,%2520and%2520any%2520third%2520party%2520is%2520permitted%2520to%2520access,%2520download,%2520copy,%2520distribute,%2520and%2520use%2520these%2520materials%2520in%2520any%2520way,%2520even%2520commercially,%2520with%2520proper%2520attribution.%2520For%2520these%2520reasons,%2520we%2520cannot%2520publish%2520previously%2520copyrighted%2520maps%2520or%2520satellite%2520images%2520created%2520using%2520proprietary%2520data,%2520such%2520as%2520Google%2520software%2520(Google%2520Maps,%2520Street%2520View,%2520and%2520Earth).%2520For%2520more%2520information,%2520see%2520our%2520copyright%2520guidelines:%2520http:%2Fjournals.plos.org%2Fplosone%2Fs%2Flicenses-and-copyright.%250b%250bWe%2520require%2520you%2520to%2520either%2520(1)%2520present%2520written%2520permission%2520from%2520the%2520copyright%2520holder%2520to%2520publish%2520these%2520figures%2520specifically%2520under%2520the%2520CC%2520BY%25204.0%2520license,%2520or%2520(2)%2520remove%2520the%2520figures%2520from%2520your%2520submission:%250b%250ba.%2520You%2520may%2520seek%2520permission%2520from%2520the%2520original%2520copyright%2520holder%2520of%2520Figure%25204%2520to%2520publish%2520the%2520content%2520specifically%2520under%2520the%2520CC%2520BY%25204.0%2520license.%2520%250b%250bWe%2520recommend%2520that%2520you%2520contact%2520the%2520original%2520copyright%2520holder%2520with%2520the%2520Content%2520Permission%2520Form%2520(http:%2Fjournals.plos.org%2Fplosone%2Fs%2Ffile%3Fid=7c09%2Fcontent-permission-form.pdf)%2520and%2520the%2520following%2520text:%250b%E2%80%9CI%2520request%2520permission%2520for%2520the%2520open-access%2520journal%2520PLOS%2520ONE%2520to%2520publish%2520XXX%2520under%2520the%2520Creative%2520Commons%2520Attribution%2520License%2520(CCAL)%2520CC%2520BY%25204.0%2520(http:%2F%2Fcreativecommons.org%2Flicenses%2Fby%2F4.0%2F).%2520Please%2520be%2520aware%2520that%2520this%2520license%2520allows%2520unrestricted%2520use%2520and%2520distribution,%2520even%2520commercially,%2520by%2520third%2520parties.%2520Please%2520reply%2520and%2520provide%2520explicit%2520written%2520permission%2520to%2520publish%2520XXX%2520under%2520a%2520CC%2520BY%2520license%2520and%2520complete%2520the%2520attached%2520form.%E2%80%9D%250b%250bPlease%2520upload%2520the%2520completed%2520Content%2520Permission%2520Form%2520or%2520other%2520proof%2520of%2520granted%2520permissions%2520as%2520an%2520%2522Other%2522%2520file%2520with%2520your%2520submission.%250b%250bIn%2520the%2520figure%2520caption%2520of%2520the%2520copyrighted%2520figure,%2520please%2520include%2520the%2520following%2520text:%2520%E2%80%9CReprinted%2520from%2520%255bref%255d%2520under%2520a%2520CC%2520BY%2520license,%2520with%2520permission%2520from%2520%255bname%2520of%2520publisher%255d,%2520original%2520copyright%2520%255boriginal%2520copyright%2520year%255d.%E2%80%9D%250b%250bb.%2520If%2520you%2520are%2520unable%2520to%2520obtain%2520permiss/1/010f019e29865dbe-902774bc-9d7d-4da2-8062-6068bc94f825-000000/PNnKSUZsy__Zcl4IdOUTkaqPrlHmA14iOezaA2RB794=258)

[Landsat: http://landsat.visibleearth.nasa.gov/](http://track.editorialmanager.com/CL0/We%2520note%2520that%2520Figure%25204%2520in%2520your%2520submission%2520contain%2520%255bmap%2Fsatellite%255d%2520images%2520which%2520may%2520be%2520copyrighted.%2520All%2520PLOS%2520content%2520is%2520published%2520under%2520the%2520Creative%2520Commons%2520Attribution%2520License%2520(CC%2520BY%25204.0),%2520which%2520means%2520that%2520the%2520manuscript,%2520images,%2520and%2520Supporting%2520Information%2520files%2520will%2520be%2520freely%2520available%2520online,%2520and%2520any%2520third%2520party%2520is%2520permitted%2520to%2520access,%2520download,%2520copy,%2520distribute,%2520and%2520use%2520these%2520materials%2520in%2520any%2520way,%2520even%2520commercially,%2520with%2520proper%2520attribution.%2520For%2520these%2520reasons,%2520we%2520cannot%2520publish%2520previously%2520copyrighted%2520maps%2520or%2520satellite%2520images%2520created%2520using%2520proprietary%2520data,%2520such%2520as%2520Google%2520software%2520(Google%2520Maps,%2520Street%2520View,%2520and%2520Earth).%2520For%2520more%2520information,%2520see%2520our%2520copyright%2520guidelines:%2520http:%2Fjournals.plos.org%2Fplosone%2Fs%2Flicenses-and-copyright.%250b%250bWe%2520require%2520you%2520to%2520either%2520(1)%2520present%2520written%2520permission%2520from%2520the%2520copyright%2520holder%2520to%2520publish%2520these%2520figures%2520specifically%2520under%2520the%2520CC%2520BY%25204.0%2520license,%2520or%2520(2)%2520remove%2520the%2520figures%2520from%2520your%2520submission:%250b%250ba.%2520You%2520may%2520seek%2520permission%2520from%2520the%2520original%2520copyright%2520holder%2520of%2520Figure%25204%2520to%2520publish%2520the%2520content%2520specifically%2520under%2520the%2520CC%2520BY%25204.0%2520license.%2520%250b%250bWe%2520recommend%2520that%2520you%2520contact%2520the%2520original%2520copyright%2520holder%2520with%2520the%2520Content%2520Permission%2520Form%2520(http:%2Fjournals.plos.org%2Fplosone%2Fs%2Ffile%3Fid=7c09%2Fcontent-permission-form.pdf)%2520and%2520the%2520following%2520text:%250b%E2%80%9CI%2520request%2520permission%2520for%2520the%2520open-access%2520journal%2520PLOS%2520ONE%2520to%2520publish%2520XXX%2520under%2520the%2520Creative%2520Commons%2520Attribution%2520License%2520(CCAL)%2520CC%2520BY%25204.0%2520(http:%2F%2Fcreativecommons.org%2Flicenses%2Fby%2F4.0%2F).%2520Please%2520be%2520aware%2520that%2520this%2520license%2520allows%2520unrestricted%2520use%2520and%2520distribution,%2520even%2520commercially,%2520by%2520third%2520parties.%2520Please%2520reply%2520and%2520provide%2520explicit%2520written%2520permission%2520to%2520publish%2520XXX%2520under%2520a%2520CC%2520BY%2520license%2520and%2520complete%2520the%2520attached%2520form.%E2%80%9D%250b%250bPlease%2520upload%2520the%2520completed%2520Content%2520Permission%2520Form%2520or%2520other%2520proof%2520of%2520granted%2520permissions%2520as%2520an%2520%2522Other%2522%2520file%2520with%2520your%2520submission.%250b%250bIn%2520the%2520figure%2520caption%2520of%2520the%2520copyrighted%2520figure,%2520please%2520include%2520the%2520following%2520text:%2520%E2%80%9CReprinted%2520from%2520%255bref%255d%2520under%2520a%2520CC%2520BY%2520license,%2520with%2520permission%2520from%2520%255bname%2520of%2520publisher%255d,%2520original%2520copyright%2520%255boriginal%2520copyright%2520year%255d.%E2%80%9D%250b%250bb.%2520If%2520you%2520are%2520unable%2520to%2520obtain%2520permiss/1/010f019e29865dbe-902774bc-9d7d-4da2-8062-6068bc94f825-000000/PNnKSUZsy__Zcl4IdOUTkaqPrlHmA14iOezaA2RB794=258)

[USGS EROS (Earth Resources Observatory and Science (EROS) Center) (public domain): http://eros.usgs.gov/#](http://track.editorialmanager.com/CL0/We%2520note%2520that%2520Figure%25204%2520in%2520your%2520submission%2520contain%2520%255bmap%2Fsatellite%255d%2520images%2520which%2520may%2520be%2520copyrighted.%2520All%2520PLOS%2520content%2520is%2520published%2520under%2520the%2520Creative%2520Commons%2520Attribution%2520License%2520(CC%2520BY%25204.0),%2520which%2520means%2520that%2520the%2520manuscript,%2520images,%2520and%2520Supporting%2520Information%2520files%2520will%2520be%2520freely%2520available%2520online,%2520and%2520any%2520third%2520party%2520is%2520permitted%2520to%2520access,%2520download,%2520copy,%2520distribute,%2520and%2520use%2520these%2520materials%2520in%2520any%2520way,%2520even%2520commercially,%2520with%2520proper%2520attribution.%2520For%2520these%2520reasons,%2520we%2520cannot%2520publish%2520previously%2520copyrighted%2520maps%2520or%2520satellite%2520images%2520created%2520using%2520proprietary%2520data,%2520such%2520as%2520Google%2520software%2520(Google%2520Maps,%2520Street%2520View,%2520and%2520Earth).%2520For%2520more%2520information,%2520see%2520our%2520copyright%2520guidelines:%2520http:%2Fjournals.plos.org%2Fplosone%2Fs%2Flicenses-and-copyright.%250b%250bWe%2520require%2520you%2520to%2520either%2520(1)%2520present%2520written%2520permission%2520from%2520the%2520copyright%2520holder%2520to%2520publish%2520these%2520figures%2520specifically%2520under%2520the%2520CC%2520BY%25204.0%2520license,%2520or%2520(2)%2520remove%2520the%2520figures%2520from%2520your%2520submission:%250b%250ba.%2520You%2520may%2520seek%2520permission%2520from%2520the%2520original%2520copyright%2520holder%2520of%2520Figure%25204%2520to%2520publish%2520the%2520content%2520specifically%2520under%2520the%2520CC%2520BY%25204.0%2520license.%2520%250b%250bWe%2520recommend%2520that%2520you%2520contact%2520the%2520original%2520copyright%2520holder%2520with%2520the%2520Content%2520Permission%2520Form%2520(http:%2Fjournals.plos.org%2Fplosone%2Fs%2Ffile%3Fid=7c09%2Fcontent-permission-form.pdf)%2520and%2520the%2520following%2520text:%250b%E2%80%9CI%2520request%2520permission%2520for%2520the%2520open-access%2520journal%2520PLOS%2520ONE%2520to%2520publish%2520XXX%2520under%2520the%2520Creative%2520Commons%2520Attribution%2520License%2520(CCAL)%2520CC%2520BY%25204.0%2520(http:%2F%2Fcreativecommons.org%2Flicenses%2Fby%2F4.0%2F).%2520Please%2520be%2520aware%2520that%2520this%2520license%2520allows%2520unrestricted%2520use%2520and%2520distribution,%2520even%2520commercially,%2520by%2520third%2520parties.%2520Please%2520reply%2520and%2520provide%2520explicit%2520written%2520permission%2520to%2520publish%2520XXX%2520under%2520a%2520CC%2520BY%2520license%2520and%2520complete%2520the%2520attached%2520form.%E2%80%9D%250b%250bPlease%2520upload%2520the%2520completed%2520Content%2520Permission%2520Form%2520or%2520other%2520proof%2520of%2520granted%2520permissions%2520as%2520an%2520%2522Other%2522%2520file%2520with%2520your%2520submission.%250b%250bIn%2520the%2520figure%2520caption%2520of%2520the%2520copyrighted%2520figure,%2520please%2520include%2520the%2520following%2520text:%2520%E2%80%9CReprinted%2520from%2520%255bref%255d%2520under%2520a%2520CC%2520BY%2520license,%2520with%2520permission%2520from%2520%255bname%2520of%2520publisher%255d,%2520original%2520copyright%2520%255boriginal%2520copyright%2520year%255d.%E2%80%9D%250b%250bb.%2520If%2520you%2520are%2520unable%2520to%2520obtain%2520permiss/1/010f019e29865dbe-902774bc-9d7d-4da2-8062-6068bc94f825-000000/PNnKSUZsy__Zcl4IdOUTkaqPrlHmA14iOezaA2RB794=258)

[Natural Earth (public domain): http://www.naturalearthdata.com/](http://track.editorialmanager.com/CL0/We%2520note%2520that%2520Figure%25204%2520in%2520your%2520submission%2520contain%2520%255bmap%2Fsatellite%255d%2520images%2520which%2520may%2520be%2520copyrighted.%2520All%2520PLOS%2520content%2520is%2520published%2520under%2520the%2520Creative%2520Commons%2520Attribution%2520License%2520(CC%2520BY%25204.0),%2520which%2520means%2520that%2520the%2520manuscript,%2520images,%2520and%2520Supporting%2520Information%2520files%2520will%2520be%2520freely%2520available%2520online,%2520and%2520any%2520third%2520party%2520is%2520permitted%2520to%2520access,%2520download,%2520copy,%2520distribute,%2520and%2520use%2520these%2520materials%2520in%2520any%2520way,%2520even%2520commercially,%2520with%2520proper%2520attribution.%2520For%2520these%2520reasons,%2520we%2520cannot%2520publish%2520previously%2520copyrighted%2520maps%2520or%2520satellite%2520images%2520created%2520using%2520proprietary%2520data,%2520such%2520as%2520Google%2520software%2520(Google%2520Maps,%2520Street%2520View,%2520and%2520Earth).%2520For%2520more%2520information,%2520see%2520our%2520copyright%2520guidelines:%2520http:%2Fjournals.plos.org%2Fplosone%2Fs%2Flicenses-and-copyright.%250b%250bWe%2520require%2520you%2520to%2520either%2520(1)%2520present%2520written%2520permission%2520from%2520the%2520copyright%2520holder%2520to%2520publish%2520these%2520figures%2520specifically%2520under%2520the%2520CC%2520BY%25204.0%2520license,%2520or%2520(2)%2520remove%2520the%2520figures%2520from%2520your%2520submission:%250b%250ba.%2520You%2520may%2520seek%2520permission%2520from%2520the%2520original%2520copyright%2520holder%2520of%2520Figure%25204%2520to%2520publish%2520the%2520content%2520specifically%2520under%2520the%2520CC%2520BY%25204.0%2520license.%2520%250b%250bWe%2520recommend%2520that%2520you%2520contact%2520the%2520original%2520copyright%2520holder%2520with%2520the%2520Content%2520Permission%2520Form%2520(http:%2Fjournals.plos.org%2Fplosone%2Fs%2Ffile%3Fid=7c09%2Fcontent-permission-form.pdf)%2520and%2520the%2520following%2520text:%250b%E2%80%9CI%2520request%2520permission%2520for%2520the%2520open-access%2520journal%2520PLOS%2520ONE%2520to%2520publish%2520XXX%2520under%2520the%2520Creative%2520Commons%2520Attribution%2520License%2520(CCAL)%2520CC%2520BY%25204.0%2520(http:%2F%2Fcreativecommons.org%2Flicenses%2Fby%2F4.0%2F).%2520Please%2520be%2520aware%2520that%2520this%2520license%2520allows%2520unrestricted%2520use%2520and%2520distribution,%2520even%2520commercially,%2520by%2520third%2520parties.%2520Please%2520reply%2520and%2520provide%2520explicit%2520written%2520permission%2520to%2520publish%2520XXX%2520under%2520a%2520CC%2520BY%2520license%2520and%2520complete%2520the%2520attached%2520form.%E2%80%9D%250b%250bPlease%2520upload%2520the%2520completed%2520Content%2520Permission%2520Form%2520or%2520other%2520proof%2520of%2520granted%2520permissions%2520as%2520an%2520%2522Other%2522%2520file%2520with%2520your%2520submission.%250b%250bIn%2520the%2520figure%2520caption%2520of%2520the%2520copyrighted%2520figure,%2520please%2520include%2520the%2520following%2520text:%2520%E2%80%9CReprinted%2520from%2520%255bref%255d%2520under%2520a%2520CC%2520BY%2520license,%2520with%2520permission%2520from%2520%255bname%2520of%2520publisher%255d,%2520original%2520copyright%2520%255boriginal%2520copyright%2520year%255d.%E2%80%9D%250b%250bb.%2520If%2520you%2520are%2520unable%2520to%2520obtain%2520permiss/1/010f019e29865dbe-902774bc-9d7d-4da2-8062-6068bc94f825-000000/PNnKSUZsy__Zcl4IdOUTkaqPrlHmA14iOezaA2RB794=258)

**Response 9:**

We thank the Editorial Office for bringing this to our attention. Figure 4 is an original figure created by the authors using QGIS. The crash locations were plotted using the X and Y coordinates obtained from the study dataset, and the figure was generated by the authors. The figure does not reproduce Google Maps, Google Earth, or other proprietary map or satellite imagery. We have reviewed the figure to ensure compliance with the PLOS ONE copyright and licensing requirements and have updated the figure caption to include the appropriate source attribution where applicable. We can remove the figure if this does not comply with the journal guidelines.

**Requirement 10:**

We note that Figure(s) 6, 8 in your submission contain copyrighted images. All PLOS content is published under the Creative Commons Attribution License (CC BY 4.0), which means that the manuscript, images, and Supporting Information files will be freely available online, and any third party is permitted to access, download, copy, distribute, and use these materials in any way, even commercially, with proper attribution. For more information, see our copyright guidelines: http://journals.plos.org/plosone/s/licenses-and-copyright.

We require you to either (1) present written permission from the copyright holder to publish these figures specifically under the CC BY 4.0 license, or (2) remove the figures from your submission:

a. You may seek permission from the original copyright holder of Figure(s) 6, 8 to publish the content specifically under the CC BY 4.0 license.

We recommend that you contact the original copyright holder with the Content Permission Form (http://journals.plos.org/plosone/s/file?id=7c09/content-permission-form.pdf) and the following text:

“I request permission for the open-access journal PLOS ONE to publish XXX under the Creative Commons Attribution License (CCAL) CC BY 4.0 (http://creativecommons.org/licenses/by/4.0/). Please be aware that this license allows unrestricted use and distribution, even commercially, by third parties. Please reply and provide explicit written permission to publish XXX under a CC BY license and complete the attached form.”

Please upload the completed Content Permission Form or other proof of granted permissions as an "Other" file with your submission.

In the figure caption of the copyrighted figure, please include the following text: “Reprinted from [ref] under a CC BY license, with permission from [name of publisher], original copyright [original copyright year].”

b. If you are unable to obtain permission from the original copyright holder to publish these figures under the CC BY 4.0 license or if the copyright holder’s requirements are incompatible with the CC BY 4.0 license, please either i) remove the figure or ii) supply a replacement figure that complies with the CC BY 4.0 license. Please check copyright information on all replacement figures and update the figure caption with source information. If applicable, please specify in the figure caption text when a figure is similar but not identical to the original image and is therefore for illustrative purposes only.

**Response 10:**

We thank the Editorial Office for bringing this to our attention. Figures 6 and 8 are original figures generated by the authors using the commercial vehicle dynamics simulation software AnalyzerPro. The figures represent the outputs of the authors' own simulation models developed for this study and are not reproduced or adapted from previously published material, software documentation, or other copyrighted sources. We have reviewed the figures to ensure compliance with the PLOS ONE copyright and licensing requirements.

**Requirement 11:**

If the reviewer comments include a recommendation to cite specific previously published works, please review and evaluate these publications to determine whether they are relevant and should be cited. There is no requirement to cite these works unless the editor has indicated otherwise.

**Response 11:**

This requirement is not applicable, as neither reviewer recommended the citation of any specific previously published works.

**Reviewer 1 Comments**

**Comment 1:**

The abstract needs to clarify the problem statements.

**Response 1:**

We sincerely thank the reviewer for the valuable comments. The abstract has been revised to explicitly highlight the high burden of run-off-road and rollover crashes on Saudi Arabia's rural highway network and the lack of locally validated, evidence-based roadside clear-zone design guidance. This revision strengthens the context of the study and clearly establishes the research gap addressed.

### **Revision to the Manuscript**

The abstract has been updated to include the following:

“Saudi Arabia's rural highway network experiences a high number of severe run-off-road and rollover crashes, yet evidence-based, locally validated roadside clear-zone design guidance remains limited. This study investigates how roadside clear-zone characteristics influence the occurrence and severity of these crashes by integrating statistical analysis of in-depth investigations of multi-fatality crash data with high-fidelity vehicle simulations.”

**Comment 2:**

The data collection of 128 only for fatal accidents, which may cause bias to the model

**Response 2:**

The dataset used in this study comprises 128 in-depth investigations of multi-fatality crashes, each crash involving more than two fatalities. Unlike routine police crash records, every case was investigated by a specialized crash investigation team from the National Road Safety Centre (NRSC), which conducted detailed field investigations at the crash site. These investigations included the systematic collection of roadway geometric characteristics, roadside conditions, vehicle information, environmental factors, physical evidence, and other engineering variables that are essential for evaluating roadside clear-zone performance. Such detailed information is not available in the national police crash database and therefore cannot be obtained from larger crash datasets.

We fully acknowledge that limiting the analysis to multi-fatality crashes introduces a severity-selection bias. Accordingly, the developed model is intended to identify factors associated with the most severe roadway departure crashes rather than to represent the entire spectrum of crash severity. This limitation has now been explicitly acknowledged in the manuscript, and we have clarified that the findings should be interpreted within the context of fatal crashes.

### **Revision to the Manuscript**

The Data Collection and Preparation section has been updated to include the following in the first paragraph (Pages 6&7, lines 29-2)

“The dataset analyzed in this study comprises 127 in-depth investigations of multi-fatality crashes, each involving more than two fatalities. For every crash, a specialized investigation team from NRSC conducted a comprehensive site investigation to document roadway geometry, roadside characteristics, vehicle damage, environmental conditions, physical evidence, and other engineering variables relevant to crash reconstruction. These in-depth investigations provide substantially richer information than routine police crash records and form the basis for the statistical and simulation analyses presented in this study. Nevertheless, because the NRSC investigation program focuses on multi-fatality crashes, the dataset represents the most severe end of the crash severity spectrum. Consequently, the estimated relationships primarily reflect factors associated with multi-fatal roadway departure crashes.”

**Comment 3:**

It is not clarified whether the 128 crashes are evenly distributed across regions or whether certain regions dominate the sample.

**Response 3:**

Although Figure 2 illustrates the regional distribution of the investigated crashes, the accompanying discussion did not explicitly address the uneven distribution of the dataset. The revised manuscript now clarifies this point as stated below.

### **Revision to the Manuscript**

The following paragraph has been added to clarify this point (page 9 lines 19-26):

“Fig 2 further demonstrates that the investigated crashes are not uniformly distributed across the administrative regions of Saudi Arabia. The Riyadh Region contributes the largest proportion of cases, while other regions are represented to varying degrees. This distribution reflects the characteristics of the NRSC in-depth crash investigation cases rather than a deliberate sampling strategy. Consequently, regional differences in traffic demand, roadway characteristics, and crash exposure should be considered when interpreting the broader applicability of the developed model, although the dataset collectively captures a diverse range of rural highway environments across the Kingdom.”

**Comment 4:**

Table 3 does not clearly differentiate between dependent and independent variables.

**Response 4:**

Table 3 has been revised to clearly indicate the dependent variable

**Comment 5:**

Adopting a fixed threshold such as 0.4 for excluding variables is considered a relatively arbitrary decision and is not based on a widely accepted statistical, clarify or add ref. from literature.

**Response 5:**

### The Pearson correlation analysis was used only as an initial screening procedure to identify potentially correlated predictors before model estimation. Rather than serving as a strict statistical exclusion criterion, this preliminary assessment was complemented by Variance Inflation Factor (VIF) diagnostics, which provide a more appropriate measure of multicollinearity within regression models. All predictors retained in the final model exhibited VIF values well below commonly accepted thresholds, confirming that multicollinearity was not a concern. The manuscript has been revised to address this point as shown below.

### **Revision to the Manuscript**

The existing paragraph has been replaced with the following (page 9 lines 1-8):

“Prior to model estimation, Pearson correlation coefficients were calculated as an initial screening tool to identify pairs of variables exhibiting moderate linear association. This preliminary assessment was intended to reduce the likelihood of introducing highly correlated predictors into the same regression model and was not used as the sole criterion for variable exclusion. Subsequently, multicollinearity among the retained variables was formally evaluated using the Variance Inflation Factor (VIF). All predictors included in the final model exhibited VIF values well below commonly accepted thresholds, confirming that multicollinearity did not materially influence the estimated regression coefficients.”

**Comment 6:**

fig. 4 is based on general visual inspection of maps, without the use of any statistical spatial techniques

**Response 6:**

We appreciate the reviewer for this insightful suggestion. The objective of Figure 4 was not to perform a spatial statistical analysis but rather to provide geographical context for the investigated crash dataset. The principal objectives of this study are to identify roadway-related factors associated with run-off-road crashes through statistical modelling and to evaluate roadside design characteristics through kinematic simulation. Accordingly, the map was included as a descriptive visualization to illustrate the geographic distribution of the investigated crashes.

**Comment 7:**

It is not clarified whether the 128 crashes are evenly distributed across regions or whether certain regions dominate the sample.

**Response 7:**

To avoid unclarity, the manuscript has been revised to explicitly describe the regional distribution of the investigated crashes, clarify that the dataset is not uniformly distributed across Saudi Arabia, and discuss the implications of this distribution for the generalizability of the study findings.

### **Revision to the Manuscript**

The following paragraph has been added to clarify this point (page 9 lines 19-26):

“Fig 2 further demonstrates that the investigated crashes are not uniformly distributed across the administrative regions of Saudi Arabia. The Riyadh Region contributes the largest proportion of cases, while other regions are represented to varying degrees. This distribution reflects the characteristics of the NRSC in-depth crash investigation cases rather than a deliberate sampling strategy. Consequently, regional differences in traffic demand, roadway characteristics, and crash exposure should be considered when interpreting the broader applicability of the developed model, although the dataset collectively captures a diverse range of rural highway environments across the Kingdom.”

**Comment 8:**

The final recommendations tend toward broad generalization at the national level (Vision 2030 and the rural road network), without clearly defining the scope of application or distinguishing between different road types or risk levels, which may limit their practical applicability.

**Response 8:**

We thank the reviewer for her valuable comment. The revised conclusion now explicitly states that the study findings are primarily applicable to high-speed, multi-lane divided rural highways, which constitute the roadway environment represented by both the investigated crash dataset and the simulation scenarios. Rather than presenting generalized national recommendations, the revised manuscript identifies the roadway characteristics for which the proposed design recommendations are most appropriate and acknowledges that additional research is required before extending these recommendations to lower-speed rural roads, urban arterials, or other roadway classes.

### **Revision to the Manuscript**

The last paragraph of the conclusion has been replaced with the following paragraph (page 19 lines 9-19):

“The findings of this study are primarily applicable to high-speed, multi-lane divided rural highways with posted speed limits between 100 and 140 km/h, which represent the roadway environments examined in both the in-depth crash dataset and the kinematic simulation scenarios. Based on the combined results of the statistical analysis and vehicle dynamics simulations, wider clear zones, flatter embankment slopes where feasible, appropriate soil compaction, and effective speed management were consistently associated with improved roadside safety performance. However, because the regression analysis is observational and the simulation scenarios represent a defined set of roadway and vehicle conditions, these findings should not be interpreted as definitive evidence of causality. Instead, they should be considered practical recommendations that warrant further validation using larger and more diverse crash datasets, additional roadway environments, and broader simulation scenarios before widespread implementation.”

**Reviewer 2 Comments**

**Comment 1:**

The manuscript uses terms such as run-off-road crash and rollover crash. These should be clearly defined in the data or methodology section.

**Response 1:**

We thank the reviewer for this valuable suggestion. The definitions of run-off-road (ROR) crash and rollover crash have been added to the Introduction, based on established definitions from NHTSA and the AASHTO Roadside Design Guide. This improves clarity and ensures consistent terminology throughout the manuscript.

### **Revision to the Manuscript**

The following sentences have been added to the introduction (page 3 lines 15-20):

“For the purposes of this study, a run-off-road (ROR) crash is defined as a crash in which a vehicle departs the travel lane and encroaches onto the roadside, potentially striking roadside features or traversing roadside terrain. A rollover crash is defined as a crash in which the vehicle rotates onto its side or roof about its longitudinal axis. Although rollover crashes frequently occur following a run-off-road event, they are treated as a separate crash outcome in this study.”

**Comment 2:**

It is not fully clear whether the same dataset of 128 multi-fatality crashes was used for this model or whether another dataset was used. Authors should clearly explain the dataset, including the number of run-off-road and non-run-off-road cases

**Response 2:**

We appreciate the reviewer for highlighting this point. The manuscript has been revised to clarify that the binary logistic regression model was developed using the same dataset of 128 in-depth investigated multi-fatality crashes described in Section 3. Within this dataset, 38 crashes were classified as run-off-road crashes and 90 as non-run-off-road crashes. No additional dataset was used for model development.

### **Revision to the Manuscript**

The following sentences have been added to the manuscript (page 11 lines 6-10):

“The binary logistic regression model was developed using the same dataset of 127 in-depth investigated multi-fatality crashes described in Section 3. Of these, 38 crashes (29.7%) were classified as run-off-road crashes (coded as 1), while the remaining 90 crashes (70.3%) represented other crash types (coded as 0). No additional dataset was used for model estimation.”

**Comment 3:**

The title and framing refer to both run-off-road and rollover crashes. However, the logistic regression model uses run-off-road crash as the dependent variable. Authors should explain this choice.

**Response 3:**

### The manuscript has been revised to clarify that the two crash types are addressed through complementary analytical approaches. Run-off-road crash was selected as the dependent variable because it was consistently coded for all 128 investigated crashes, enabling reliable regression modelling. In contrast, rollover was not consistently recorded as a binary outcome across all crash reports and therefore could not be modelled statistically. Instead, rollover behaviour is investigated through the vehicle dynamics simulation component of the study.

### **Revision to the Manuscript**

The following sentences were added to the manuscript at page 8 lines 11-15:

“Although both run-off-road and rollover crashes are addressed in this study, the regression model uses run-off-road crash as the dependent variable because this outcome was consistently recorded for all investigated crashes. Rollover was not uniformly documented as a standalone binary variable and therefore could not be reliably modelled using logistic regression. Instead, rollover behaviour is examined through the kinematic simulation scenarios”.

**Comment 4:**

There appears to be a typographical issue in the logistic regression formula in Section 4.1. The equation should be carefully revised and presented in standard format and numbered.

**Response 4:**

### We apologize for the error. The logistic regression equation has been corrected, reformatted using the standard logit expression, and assigned an equation number for consistency and ease of reference throughout the manuscript.

### **Revision to the Manuscript**

Replace the existing equation with:

ln⁡(P1−P)=β0+β1X1+β2X2+⋯+βiXi\ln\left(\frac{P}{1-P}\right)=\beta_0+\beta_1X_1+\beta_2X_2+\cdots+\beta_iX_iln(1−PP)=β0+β1X1+β2X2+⋯+βiXi

where *P* is the probability of a run-off-road crash.

**Comment 5:**

Table 4 appears to be copied from the statistical software output. The table should be reformatted into a cleaner and more professional manuscript-style table.

**Response 5:**

### Table 4 has been completely revised as requested

**Comment 6:**

Authors should provide clearer justification for why binary logistic regression was selected. Other approaches such as random forest, gradient boosting, SVM, or penalized logistic regression could also be considered

**Response 6:**

### The manuscript has been revised to better justify the selection of binary logistic regression. This approach was considered appropriate because the outcome variable is binary, the dataset is relatively small (127 observations with 38 positive events), and the study aims to produce interpretable results that can directly support engineering decision-making. Binary logistic regression provides easily interpretable odds ratios and has been widely used in comparable traffic safety studies. We have also acknowledged that machine learning methods may be explored in future studies using larger datasets.

### **Revision to the Manuscript**

The following paragraph has been added in page 11 lines 15-22:

“Binary logistic regression was selected because the dependent variable is binary and the study seeks to identify interpretable relationships between roadway characteristics and run-off-road crash occurrence. Given the relatively modest sample size and the objective of producing engineering-oriented guidance, logistic regression provides a robust and transparent modelling framework. While machine learning techniques may offer improved predictive performance with larger datasets, they generally require substantially more observations and often provide less interpretable results. Future research using larger crash databases may explore these approaches for comparison.”

**Comment 7:**

The manuscript provides useful recommendations related to clear-zone widening, soil compaction, slope moderation, and speed management. However, the conclusions should avoid implying strong causal effects because the regression analysis is observational and the simulation scenarios are limited. The authors should frame the recommendations as evidence-informed findings that require further validation using larger and more diverse crash datasets.

**Response 7:**

### We agree that the conclusions should clearly distinguish between evidence-supported recommendations and causal inference. The manuscript has been revised to present the proposed design recommendations as evidence-informed guidance derived from the combined findings of the observational crash analysis and the simulation study, rather than as definitive causal conclusions. We have also emphasized that these recommendations should be further validated using larger and more diverse crash datasets before broader implementation.

### **Revision to the Manuscript**

The last paragraph of the conclusion has been replaced with the following paragraph (page 19lines 9-20):

“The findings of this study are primarily applicable to high-speed, multi-lane divided rural highways with posted speed limits between 100 and 140 km/h, which represent the roadway environments examined in both the in-depth crash dataset and the kinematic simulation scenarios. Based on the combined results of the statistical analysis and vehicle dynamics simulations, wider clear zones, flatter embankment slopes where feasible, appropriate soil compaction, and effective speed management were consistently associated with improved roadside safety performance. However, because the regression analysis is observational and the simulation scenarios represent a defined set of roadway and vehicle conditions, these findings should not be interpreted as definitive evidence of causality. Instead, they should be considered practical recommendations that warrant further validation using larger and more diverse crash datasets, additional roadway environments, and broader simulation scenarios before widespread implementation.”
