# Supplementary material for: Evidence-based roadside clear-zone design for mitigating run-off-road and rollover crashes on rural highways in Saudi Arabia
Source: PLoS One. 2026 Sep 16;21(9):e0357284. doi: 10.1371/journal.pone.0357284 (PMC13581012; doi:10.1371/journal.pone.0357284)
Supplement: Peer review history

# Peer review: Decision Letter 0

**DOI:** 10.1371/journal.pone.0357284.r001

**Authors:** Iman Aghayan

14 May 2026

Dear Dr. Alarifi,

Please submit your revised manuscript by Jun 28 2026 11:59PM. If you will need more time than this to complete your revisions, please reply to this message or contact the journal office at plosone@plos.org. When you're ready to submit your revision, log on to https://www.editorialmanager.com/pone/ and select the 'Submissions Needing Revision' folder to locate your manuscript file.

- A letter that responds to each point raised by the academic editor and reviewer(s). You should upload this letter as a separate file labeled 'Response to Reviewers'.
- A marked-up copy of your manuscript that highlights changes made to the original version. You should upload this as a separate file labeled 'Revised Manuscript with Track Changes'.
- An unmarked version of your revised paper without tracked changes. You should upload this as a separate file labeled 'Manuscript'.

If you would like to make changes to your financial disclosure, please include your updated statement in your cover letter. Guidelines for resubmitting your figure files are available below the reviewer comments at the end of this letter.

If applicable, we recommend that you deposit your laboratory protocols in protocols.io to enhance the reproducibility of your results. Protocols.io assigns your protocol its own identifier (DOI) so that it can be cited independently in the future. For instructions see: https://journals.plos.org/plosone/s/submission-guidelines#loc-laboratory-protocols. Additionally, PLOS ONE offers an option for publishing peer-reviewed Lab Protocol articles, which describe protocols hosted on protocols.io. Read more information on sharing protocols at https://plos.org/protocols?utm_medium=editorial-email&utm_source=authorletters&utm_campaign=protocols.

As the corresponding author, your ORCID iD is verified in the submission system and will appear in the published article. PLOS supports the use of ORCID, and we encourage all coauthors to register for an ORCID iD and use it as well. Please encourage your coauthors to verify their ORCID iD within the submission system before final acceptance, as unverified ORCID iDs will not appear in the published article. *Only*  the individual author can complete the verification step; PLOS staff *cannot*  verify ORCID iDs on behalf of authors.

We look forward to receiving your revised manuscript.

Kind regards,

Iman Aghayan, Ph.D.

Academic Editor

PLOS One

Journal Requirements:

When submitting your revision, we need you to address these additional requirements.

1. Please ensure that your manuscript meets PLOS ONE's style requirements, including those for file naming. The PLOS ONE style templates can be found at

https://journals.plos.org/plosone/s/file?id=wjVg/PLOSOne_formatting_sample_main_body.pdf and https://journals.plos.org/plosone/s/file?id=ba62/PLOSOne_formatting_sample_title_authors_affiliations.pdf

2. Please note that PLOS One has specific guidelines on code sharing for submissions in which author-generated code underpins the findings in the manuscript. In these cases, we expect all author-generated code to be made available without restrictions upon publication of the work. Please review our guidelines at https://journals.plos.org/plosone/s/materials-and-software-sharing#loc-sharing-code and ensure that your code is shared in a way that follows best practice and facilitates reproducibility and reuse.

3. We note that you have indicated that there are restrictions to data sharing for this study. PLOS only allows data to be available upon request if there are legal or ethical restrictions on sharing data publicly. For more information on unacceptable data access restrictions, please see http://journals.plos.org/plosone/s/data-availability#loc-unacceptable-data-access-restrictions.

Before we proceed with your manuscript, please address the following prompts:

a) If there are ethical or legal restrictions on sharing a de-identified data set, please explain them in detail (e.g., data contain potentially identifying or sensitive patient information, data are owned by a third-party organization, etc.) and who has imposed them (e.g., a Research Ethics Committee or Institutional Review Board, etc.). Please also provide contact information for a data access committee, ethics committee, or other institutional body to which data requests may be sent.

b) If there are no restrictions, please upload the minimal anonymized data set necessary to replicate your study findings to a stable, public repository and provide us with the relevant URLs, DOIs, or accession numbers. For a list of recommended repositories, please see

https://journals.plos.org/plosone/s/recommended-repositories. You also have the option of uploading the data as Supporting Information files, but we would recommend depositing data directly to a data repository if possible.

We will update your Data Availability statement on your behalf to reflect the information you provide.

4. Please provide a complete Data Availability Statement in the submission form, ensuring you include all necessary access information or a reason for why you are unable to make your data freely accessible. If your research concerns only data provided within your submission, please write "All data are in the manuscript and/or supporting information files" as your Data Availability Statement.

5. Thank you for stating the following in the Acknowledgments Section of your manuscript:

The authors would like to thank the National Transport Safety Center for providing the crash datasets and for helping in our understanding of the datasets. In addition, the authors would like to thank the Ongoing Research Funding Program, (ORFFT-2025-081-3), King Saud University, Riyadh, Saudi Arabia for financial support. Lastly, we would like to also thank Konstantia Kontodimou for her help.

We note that you have provided funding information that is not currently declared in your Funding Statement. However, funding information should not appear in the Acknowledgments section or other areas of your manuscript. We will only publish funding information present in the Funding Statement section of the online submission form.

Please remove any funding-related text from the manuscript and let us know how you would like to update your Funding Statement. Currently, your Funding Statement reads as follows:

The author(s) received no specific funding for this work.

Please include your amended statements within your cover letter; we will change the online submission form on your behalf.

6. Please update your submission to use the PLOS LaTeX template. The template and more information on our requirements for LaTeX submissions can be found at http://journals.plos.org/plosone/s/latex.

7. Please amend your authorship list in your manuscript file to include author Saif A. Alarifi.

8. Please amend the manuscript submission data (via Edit Submission) to include author Saif Alarifi.

9. We note that Figure 4 in your submission contain [map/satellite] images which may be copyrighted. All PLOS content is published under the Creative Commons Attribution License (CC BY 4.0), which means that the manuscript, images, and Supporting Information files will be freely available online, and any third party is permitted to access, download, copy, distribute, and use these materials in any way, even commercially, with proper attribution. For these reasons, we cannot publish previously copyrighted maps or satellite images created using proprietary data, such as Google software (Google Maps, Street View, and Earth). For more information, see our copyright guidelines: http://journals.plos.org/plosone/s/licenses-and-copyright.

We require you to either (1) present written permission from the copyright holder to publish these figures specifically under the CC BY 4.0 license, or (2) remove the figures from your submission:

a. You may seek permission from the original copyright holder of Figure 4 to publish the content specifically under the CC BY 4.0 license.

We recommend that you contact the original copyright holder with the Content Permission Form (http://journals.plos.org/plosone/s/file?id=7c09/content-permission-form.pdf) and the following text:

“I request permission for the open-access journal PLOS ONE to publish XXX under the Creative Commons Attribution License (CCAL) CC BY 4.0 (http://creativecommons.org/licenses/by/4.0/). Please be aware that this license allows unrestricted use and distribution, even commercially, by third parties. Please reply and provide explicit written permission to publish XXX under a CC BY license and complete the attached form.”

Please upload the completed Content Permission Form or other proof of granted permissions as an "Other" file with your submission.

In the figure caption of the copyrighted figure, please include the following text: “Reprinted from [ref] under a CC BY license, with permission from [name of publisher], original copyright [original copyright year].”

b. If you are unable to obtain permission from the original copyright holder to publish these figures under the CC BY 4.0 license or if the copyright holder’s requirements are incompatible with the CC BY 4.0 license, please either i) remove the figure or ii) supply a replacement figure that complies with the CC BY 4.0 license. Please check copyright information on all replacement figures and update the figure caption with source information. If applicable, please specify in the figure caption text when a figure is similar but not identical to the original image and is therefore for illustrative purposes only.

The following resources for replacing copyrighted map figures may be helpful:

USGS National Map Viewer (public domain): http://viewer.nationalmap.gov/viewer/

The Gateway to Astronaut Photography of Earth (public domain): http://eol.jsc.nasa.gov/sseop/clickmap/

Maps at the CIA (public domain): https://www.cia.gov/library/publications/the-world-factbook/index.html and https://www.cia.gov/library/publications/cia-maps-publications/index.html

NASA Earth Observatory (public domain): http://earthobservatory.nasa.gov/

Landsat: http://landsat.visibleearth.nasa.gov/

USGS EROS (Earth Resources Observatory and Science (EROS) Center) (public domain): http://eros.usgs.gov/#

Natural Earth (public domain): http://www.naturalearthdata.com/

10. We note that Figure(s) 6, 8 in your submission contain copyrighted images. All PLOS content is published under the Creative Commons Attribution License (CC BY 4.0), which means that the manuscript, images, and Supporting Information files will be freely available online, and any third party is permitted to access, download, copy, distribute, and use these materials in any way, even commercially, with proper attribution. For more information, see our copyright guidelines: http://journals.plos.org/plosone/s/licenses-and-copyright.

We require you to either (1) present written permission from the copyright holder to publish these figures specifically under the CC BY 4.0 license, or (2) remove the figures from your submission:

a. You may seek permission from the original copyright holder of Figure(s) 6, 8 to publish the content specifically under the CC BY 4.0 license.

We recommend that you contact the original copyright holder with the Content Permission Form (http://journals.plos.org/plosone/s/file?id=7c09/content-permission-form.pdf) and the following text:

“I request permission for the open-access journal PLOS ONE to publish XXX under the Creative Commons Attribution License (CCAL) CC BY 4.0 (http://creativecommons.org/licenses/by/4.0/). Please be aware that this license allows unrestricted use and distribution, even commercially, by third parties. Please reply and provide explicit written permission to publish XXX under a CC BY license and complete the attached form.”

Please upload the completed Content Permission Form or other proof of granted permissions as an "Other" file with your submission.

In the figure caption of the copyrighted figure, please include the following text: “Reprinted from [ref] under a CC BY license, with permission from [name of publisher], original copyright [original copyright year].”

b. If you are unable to obtain permission from the original copyright holder to publish these figures under the CC BY 4.0 license or if the copyright holder’s requirements are incompatible with the CC BY 4.0 license, please either i) remove the figure or ii) supply a replacement figure that complies with the CC BY 4.0 license. Please check copyright information on all replacement figures and update the figure caption with source information. If applicable, please specify in the figure caption text when a figure is similar but not identical to the original image and is therefore for illustrative purposes only.

11. If the reviewer comments include a recommendation to cite specific previously published works, please review and evaluate these publications to determine whether they are relevant and should be cited. There is no requirement to cite these works unless the editor has indicated otherwise.

[Note: HTML markup is below. Please do not edit.]

Reviewers' comments:

Reviewer's Responses to Questions

**Comments to the Author**

1. Is the manuscript technically sound, and do the data support the conclusions?

Reviewer #1: Yes

Reviewer #2: Yes

**********

2. Has the statistical analysis been performed appropriately and rigorously?

Reviewer #1: Yes

Reviewer #2: Yes

**********

3. Have the authors made all data underlying the findings in their manuscript fully available?

The PLOS Data policy

Reviewer #1: Yes

Reviewer #2: No

**********

4. Is the manuscript presented in an intelligible fashion and written in standard English?

Reviewer #1: Yes

Reviewer #2: Yes

**********

Reviewer #1: 1. The abstract need to clerify the problem statemnts.

2. The data collection 128 only for fata accidents, which may cause bias to model

3. It is not clarified whether the 128 crashes are evenly distributed across regions or whether certain regions dominate the sample

4. table 3 does not clearly differentiate between dependent and independent variables

5. adopting a fixed threshold such as 0.4 for excluding variables is considered a relatively arbitrary decision and is not based on a widely accepted statistical, clerify or add ref. from literature.

6. fig. 4 is based on general visual inspection of maps, without the use of any statistical spatial techniques

7. It is not clarified whether the 128 crashes are evenly distributed across regions or whether certain regions dominate the sample.

8. The final recommendations tend toward broad generalization at the national level (Vision 2030 and the rural road network), without clearly defining the scope of application or distinguishing between different road types or risk levels, which may limit their practical applicability

-----------------

Reviewer #2: This study explores roadside clear-zone design and its potential role in reducing run-off-road and rollover crashes on rural highways in Saudi Arabia using both statistical modeling and simulation analysis. This is an important issue and is worth studying, especially given the safety concerns associated with rural highway crashes. The combination of statistical modeling and simulation makes the study potentially valuable. Following are my comments:

1. The manuscript uses terms such as run-off-road crash and rollover crash. Although these terms may be familiar to transportation safety researchers, they should be clearly defined in the data or methodology section. This would help readers who may not be familiar with these crash types and would also improve the clarity of the study scope.

2. In Section 4.1, the manuscript states that the binary dependent variable is defined as “run-off-road crash (1) versus other crashes (0).” However, it is not fully clear whether the same dataset of 128 multi-fatality crashes was used for this model or whether another dataset including both run-off-road and non-run-off-road crashes was used. The authors should clearly explain the dataset used for model estimation, including the number of run-off-road and non-run-off-road cases.

3. The title and framing of the paper refer to both run-off-road and rollover crashes. However, the logistic regression model appears to use run-off-road crash as the dependent variable. The authors should explain why run-off-road crash was selected as the modeling outcome rather than rollover crash. If the data allow, the authors may consider estimating two separate models: one for run-off-road crashes and another for rollover crashes.

4. There appears to be a typographical issue in the logistic regression formula in Section 4.1. The equation should be carefully revised and presented in a standard format. In addition, the equation should be numbered so it can be clearly referenced in the text.

5. Table 4 appears to be copied from the statistical software output. The table should be reformatted into a cleaner and more professional manuscript-style table.

6. The authors should provide a clearer justification for why binary logistic regression was selected as the final modeling approach. Since the study uses a relatively small dataset, and since other modeling approaches such as random forest, gradient boosting, support vector machines, or penalized logistic regression could also be considered for binary classification, the authors should explain why binary logistic regression is the most appropriate method for this study. For example, if the choice was based on interpretability, ease of explaining odds ratios, suitability for small samples, or consistency with prior crash-severity studies, this should be clearly stated. The authors should also discuss whether more complex machine-learning models were considered and why they were not used.

7. The manuscript provides useful recommendations related to clear-zone widening, soil compaction, slope moderation, and speed management. However, the conclusions should avoid implying strong causal effects because the regression analysis is observational and the simulation scenarios are limited. The authors should frame the recommendations as evidence-informed findings that require further validation using larger and more diverse crash datasets.

**********

what does this mean?). If published, this will include your full peer review and any attached files.

If you choose “no”, your identity will remain anonymous but your review may still be made public.

**Do you want your identity to be public for this peer review?**  For information about this choice, including consent withdrawal, please see our Privacy Policy

Reviewer #1: **Yes:** Zainab Alkaissi

Reviewer #2: No

**********

[NOTE: If reviewer comments were submitted as an attachment file, they will be attached to this email and accessible via the submission site. Please log into your account, locate the manuscript record, and check for the action link "View Attachments". If this link does not appear, there are no attachment files.]

To ensure your figures meet our technical requirements, please review our figure guidelines: https://journals.plos.org/plosone/s/figures

You may also use PLOS’s free figure tool, NAAS, to help you prepare publication quality figures: https://journals.plos.org/plosone/s/figures#loc-tools-for-figure-preparation.

NAAS will assess whether your figures meet our technical requirements by comparing each figure against our figure specifications.

# Author comment: Author response to Decision Letter 1

**DOI:** 10.1371/journal.pone.0357284.r002

14 Jul 2026

Response to Reviewers

Manuscript ID: PONE-D-26-08573

Title: Evidence-Based Roadside Clear-Zone Design for Mitigating Run-Off-Road and Rollover Crashes on Rural Highways in Saudi Arabia

Journal: PLOS ONE

Corresponding Author: Dr. Saif Alarifi

Dear Dr. Iman Aghayan,

We sincerely thank the reviewers for their thorough and constructive feedback on our manuscript. We have carefully addressed all comments and revised the manuscript accordingly. Below, we provide a point-by-point response to each comment, indicating where changes have been made in the revised manuscript.

Journal requirements

When submitting your revision, we need you to address these additional requirements.

Requirement 1:

Please ensure that your manuscript meets PLOS ONE's style requirements, including those for file naming. The PLOS ONE style templates can be found at

https://journals.plos.org/plosone/s/file?id=wjVg/PLOSOne_formatting_sample_main_body.pdf and https://journals.plos.org/plosone/s/file?id=ba62/PLOSOne_formatting_sample_title_authors_affiliations.pdf

Response 1:

We thank the Editorial Office for this observation. The manuscript has been revised to fully comply with the PLOS ONE formatting guidelines, including the prescribed manuscript structure, headings, tables, figures, references, and file naming conventions.

Requirement 2:

Please note that PLOS One has specific guidelines on code sharing for submissions in which author-generated code underpins the findings in the manuscript. In these cases, we expect all author-generated code to be made available without restrictions upon publication of the work. Please review our guidelines at https://journals.plos.org/plosone/s/materials-and-software-sharing#loc-sharing-code and ensure that your code is shared in a way that follows best practice and facilitates reproducibility and reuse.

Response 2:

This study did not involve the development of any author-generated software or custom computer code. Statistical analyses were performed using IBM SPSS Statistics, geographic visualization was prepared using QGIS, and vehicle simulations were conducted using the commercial software AnalyzerPro. Therefore, no custom code underpins the findings of this study.

Requirement 3:

We note that you have indicated that there are restrictions to data sharing for this study. PLOS only allows data to be available upon request if there are legal or ethical restrictions on sharing data publicly. For more information on unacceptable data access restrictions, please see http://journals.plos.org/plosone/s/data-availability#loc-unacceptable-data-access-restrictions.

Before we proceed with your manuscript, please address the following prompts:

a) If there are ethical or legal restrictions on sharing a de-identified data set, please explain them in detail (e.g., data contain potentially identifying or sensitive patient information, data are owned by a third-party organization, etc.) and who has imposed them (e.g., a Research Ethics Committee or Institutional Review Board, etc.). Please also provide contact information for a data access committee, ethics committee, or other institutional body to which data requests may be sent.

b) If there are no restrictions, please upload the minimal anonymized data set necessary to replicate your study findings to a stable, public repository and provide us with the relevant URLs, DOIs, or accession numbers. For a list of recommended repositories, please see

https://journals.plos.org/plosone/s/recommended-repositories. You also have the option of uploading the data as Supporting Information files, but we would recommend depositing data directly to a data repository if possible.

We will update your Data Availability statement on your behalf to reflect the information you provide.

Response 3:

We thank the Editorial Office for this important request. The dataset used in this study is owned by the National Road Safety Centre (NRSC). The authors have obtained permission from the data owner to provide the dataset to the PLOS ONE Editorial Office and reviewers for the purpose of evaluating this manuscript. However, the authors are not authorized to make the dataset publicly available or deposit it in a public repository, as public disclosure is restricted by the data owner. We respectfully request that the dataset be treated as confidential and used solely for editorial and peer-review purposes.

Requirement 4:

4. Please provide a complete Data Availability Statement in the submission form, ensuring you include all necessary access information or a reason for why you are unable to make your data freely accessible. If your research concerns only data provided within your submission, please write "All data are in the manuscript and/or supporting information files" as your Data Availability Statement.

Response 4:

We thank the Editorial Office for this request. The Data Availability Statement has been revised to clearly describe the data access restrictions, the reason the dataset cannot be made publicly available, and the process by which qualified researchers may request access from the data owner.

Requirement 5:

Thank you for stating the following in the Acknowledgments Section of your manuscript:

The authors would like to thank the National Transport Safety Center for providing the crash datasets and for helping in our understanding of the datasets. In addition, the authors would like to thank the Ongoing Research Funding Program, (ORFFT-2025-081-3), King Saud University, Riyadh, Saudi Arabia for financial support. Lastly, we would like to also thank Konstantia Kontodimou for her help.

We note that you have provided funding information that is not currently declared in your Funding Statement. However, funding information should not appear in the Acknowledgments section or other areas of your manuscript. We will only publish funding information present in the Funding Statement section of the online submission form.

Please remove any funding-related text from the manuscript and let us know how you would like to update your Funding Statement. Currently, your Funding Statement reads as follows:

The author(s) received no specific funding for this work.

Please include your amended statements within your cover letter; we will change the online submission form on your behalf.

Response 5:

We thank the Editorial Office for identifying this inconsistency. The funding information has been removed from the Acknowledgments section and transferred to the Funding Statement. The Funding Statement has been updated to accurately reflect the financial support received for this study.

Requirement 6:

Please update your submission to use the PLOS LaTeX template. The template and more information on our requirements for LaTeX submissions can be found at http://journals.plos.org/plosone/s/latex.

Response 6:

We thank the Editorial Office for this note. This requirement is not applicable, as the manuscript was prepared and submitted using Microsoft Word rather than LaTeX. The revised manuscript has been prepared in accordance with the PLOS ONE Microsoft Word formatting guidelines.

Requirement 7:

Please amend your authorship list in your manuscript file to include author Saif A. Alarifi.

Response 7:

Done

Requirement 8:

Please amend the manuscript submission data (via Edit Submission) to include author Saif Alarifi.

Response 8:

Done

Requirement 9:

We note that Figure 4 in your submission contain [map/satellite] images which may be copyrighted. All PLOS content is published under the Creative Commons Attribution License (CC BY 4.0), which means that the manuscript, images, and Supporting Information files will be freely available online, and any third party is permitted to access, download, copy, distribute, and use these materials in any way, even commercially, with proper attribution. For these reasons, we cannot publish previously copyrighted maps or satellite images created using proprietary data, such as Google software (Google Maps, Street View, and Earth). For more information, see our copyright guidelines: http://journals.plos.org/plosone/s/licenses-and-copyright.

We require you to either (1) present written permission from the copyright holder to publish these figures specifically under the CC BY 4.0 license, or (2) remove the figures from your submission:

a. You may seek permission from the original copyright holder of Figure 4 to publish the content specifically under the CC BY 4.0 license.

We recommend that you contact the original copyright holder with the Content Permission Form (http://journals.plos.org/plosone/s/file?id=7c09/content-permission-form.pdf) and the following text:

“I request permission for the open-access journal PLOS ONE to publish XXX under the Creative Commons Attribution License (CCAL) CC BY 4.0 (http://creativecommons.org/licenses/by/4.0/). Please be aware that this license allows unrestricted use and distribution, even commercially, by third parties. Please reply and provide explicit written permission to publish XXX under a CC BY license and complete the attached form.”

Please upload the completed Content Permission Form or other proof of granted permissions as an "Other" file with your submission.

In the figure caption of the copyrighted figure, please include the following text: “Reprinted from [ref] under a CC BY license, with permission from [name of publisher], original copyright [original copyright year].”

b. If you are unable to obtain permission from the original copyright holder to publish these figures under the CC BY 4.0 license or if the copyright holder’s requirements are incompatible with the CC BY 4.0 license, please either i) remove the figure or ii) supply a replacement figure that complies with the CC BY 4.0 license. Please check copyright information on all replacement figures and update the figure caption with source information. If applicable, please specify in the figure caption text when a figure is similar but not identical to the original image and is therefore for illustrative purposes only.

The following resources for replacing copyrighted map figures may be helpful:

USGS National Map Viewer (public domain): http://viewer.nationalmap.gov/viewer/

The Gateway to Astronaut Photography of Earth (public domain): http://eol.jsc.nasa.gov/sseop/clickmap/

Maps at the CIA (public domain): https://www.cia.gov/library/publications/the-world-factbook/index.html and https://www.cia.gov/library/publications/cia-maps-publications/index.html

NASA Earth Observatory (public domain): http://earthobservatory.nasa.gov/

Landsat: http://landsat.visibleearth.nasa.gov/

USGS EROS (Earth Resources Observatory and Science (EROS) Center) (public domain): http://eros.usgs.gov/#

Natural Earth (public domain): http://www.naturalearthdata.com/

Response 9:

We thank the Editorial Office for bringing this to our attention. Figure 4 is an original figure created by the authors using QGIS. The crash locations were plotted using the X and Y coordinates obtained from the study dataset, and the figure was generated by the authors. The figure does not reproduce Google Maps, Google Earth, or other proprietary map or satellite imagery. We have reviewed the figure to ensure compliance with the PLOS ONE copyright and licensing requirements and have updated the figure caption to include the appropriate source attribution where applicable. We can remove the figure if this does not comply with the journal guidelines.

Requirement 10:

We note that Figure(s) 6, 8 in your submission contain copyrighted images. All PLOS content is published under the Creative Commons Attribution License (CC BY 4.0), which means that the manuscript, images, and Supporting Information files will be freely available online, and any third party is permitted to access, download, copy, distribute, and use these materials in any way, even commercially, with proper attribution. For more information, see our copyright guidelines: http://journals.plos.org/plosone/s/licenses-and-copyright.

We require you to either (1) present written permission from the copyright holder to publish these figures specifically under the CC BY 4.0 license, or (2) remove the figures from your submission:

a. You may seek permission from the original copyright holder of Figure(s) 6, 8 to publish the content specifically under the CC BY 4.0 license.

We recommend that you contact the original copyright holder with the Content Permission Form (http://journals.plos.org/plosone/s/file?id=7c09/content-permission-form.pdf) and the following text:

“I request permission for the open-access journal PLOS ONE to publish XXX under the Creative Commons Attribution License (CCAL) CC BY 4.0 (http://creativecommons.org/licenses/by/4.0/). Please be aware that this license allows unrestricted use and distribution, even commercially, by third parties. Please reply and provide explicit written permission to publish XXX under a CC BY license and complete the attached form.”

Please upload the completed Content Permission Form or other proof of granted permissions as an "Other" file with your submission.

In the figure caption of the copyrighted figure, please include the following text: “Reprinted from [ref] under a CC BY license, with permission from [name of publisher], original copyright [original copyright year].”

b. If you are unable to obtain permission from the original copyright holder to publish these figures under the CC BY 4.0 license or if the copyright holder’s requirements are incompatible with the CC BY 4.0 license, please either i) remove the figure or ii) supply a replacement figure that complies with the CC BY 4.0 license. Please check copyright information on all replacement figures and update the figure caption with source information. If applicable, please specify in the figure caption text when a figure is similar but not identical to the original image and is therefore for illustrative purposes only.

Response 10:

We thank the Editorial Office for bringing this to our attention. Figures 6 and 8 are original figures generated by the authors using the commercial vehicle dynamics simulation software AnalyzerPro. The figures represent the outputs of the authors' own simulation models developed for this study and are not reproduced or adapted from previously published material, software documentation, or other copyrighted sources. We have reviewed the figures to ensure compliance with the PLOS ONE copyright and licensing requirements.

Requirement 11:

If the reviewer comments include a recommendation to cite specific previously published works, please review and evaluate these publications to determine whether they are relevant and should be cited. There is no requirement to cite these works unless the editor has indicated otherwise.

Response 11:

This requirement is not applicable, as neither reviewer recommended the citation of any specific previously published works.

Reviewer 1 Comments

Comment 1:

The abstract needs to clarify the problem statements.

Response 1:

We sincerely thank the reviewer for the valuable comments. The abstract has been revised to explicitly highlight the high burden of run-off-road and rollover crashes on Saudi Arabia's rural highway network and the lack of locally validated, evidence-based roadside clear-zone design guidance. This revision strengthens the context of the study and clearly establishes the research gap addressed.

Revision to the Manuscript

The abstract has been updated to include the following:

“Saudi Arabia's rural highway network experiences a high number of severe run-off-road and rollover crashes, yet evidence-based, locally validated roadside clear-zone design guidance remains limited. This study investigates how roadside clear-zone characteristics influence the occurrence and severity of these crashes by integrating statistical analysis of in-depth investigations of multi-fatality crash data with high-fidelity vehicle simulations.”

Comment 2:

The data collection of 128 only for fatal accidents, which may cause bias to the model

Response 2:

The dataset used in this study comprises 128 in-depth investigations of multi-fatality crashes, each crash involving more than two fatalities. Unlike routine police crash records, every case was investigated by

Attachment Submitted filename: Response to Reviewers.docx

# Peer review: Decision Letter 1

**DOI:** 10.1371/journal.pone.0357284.r003

**Authors:** Iman Aghayan

17 Aug 2026

Evidence-Based Roadside Clear-Zone Design for Mitigating Run-Off-Road and Rollover Crashes on Rural Highways in Saudi Arabia

PONE-D-26-08573R1

Dear Dr. Alarifi,

We’re pleased to inform you that your manuscript has been judged scientifically suitable for publication and will be formally accepted for publication once it meets all outstanding technical requirements.

Within one week, you’ll receive an e-mail detailing the required amendments. When these have been addressed, you’ll receive a formal acceptance letter and your manuscript will be scheduled for publication.

An invoice will be generated when your article is formally accepted. Please note, if your institution has a publishing partnership with PLOS and your article meets the relevant criteria, all or part of your publication costs will be covered. Please make sure your user information is up-to-date by logging into Editorial Manager at Editorial Manager® and clicking the ‘Update My Information' link at the top of the page. For questions related to billing, please contact billing support.

If your institution or institutions have a press office, please notify them about your upcoming paper to help maximize its impact. If they’ll be preparing press materials, please inform our press team as soon as possible -- no later than 48 hours after receiving the formal acceptance. Your manuscript will remain under strict press embargo until 2 pm Eastern Time on the date of publication. For more information, please contact onepress@plos.org.

Kind regards,

Iman Aghayan, Ph.D.

Academic Editor

PLOS One

Additional Editor Comments (optional):

Reviewers' comments:

Reviewer's Responses to Questions

**Comments to the Author**

Reviewer #1: All comments have been addressed

Reviewer #2: All comments have been addressed

**********

2. Is the manuscript technically sound, and do the data support the conclusions?

Reviewer #1: Yes

Reviewer #2: Yes

**********

3. Has the statistical analysis been performed appropriately and rigorously?

Reviewer #1: N/A

Reviewer #2: Yes

**********

4. Have the authors made all data underlying the findings in their manuscript fully available?

The PLOS Data policy

Reviewer #1: Yes

Reviewer #2: No

**********

5. Is the manuscript presented in an intelligible fashion and written in standard English?

Reviewer #1: Yes

Reviewer #2: Yes

**********

Reviewer #1: (No Response)

Reviewer #2: All of my previous comments have been addressed. The manuscript would still benefit from another round of proofreading, particularly to shorten some long sentences and improve readability.

**********

what does this mean?). If published, this will include your full peer review and any attached files.

If you choose “no”, your identity will remain anonymous but your review may still be made public.

**Do you want your identity to be public for this peer review?**  For information about this choice, including consent withdrawal, please see our Privacy Policy

Reviewer #1: **Yes:** Zainab Alkaissi

Reviewer #2: No

**********

# Editor report: Acceptance letter

**DOI:** 10.1371/journal.pone.0357284.r004

**Authors:** Iman Aghayan

PONE-D-26-08573R1

PLOS One

Dear Dr. Alarifi,

I'm pleased to inform you that your manuscript has been deemed suitable for publication in PLOS One. Congratulations! Your manuscript is now being handed over to our production team.

At this stage, our production department will prepare your paper for publication. This includes ensuring the following:

* All references, tables, and figures are properly cited

* All relevant supporting information is included in the manuscript submission,

* There are no issues that prevent the paper from being properly typeset

You will receive further instructions from the production team, including instructions on how to review your proof when it is ready. Please keep in mind that we are working through a large volume of accepted articles, so please give us a few days to review your paper and let you know the next and final steps.

Lastly, if your institution or institutions have a press office, please let them know about your upcoming paper now to help maximize its impact. If they'll be preparing press materials, please inform our press team within the next 48 hours. Your manuscript will remain under strict press embargo until 2 pm Eastern Time on the date of publication. For more information, please contact onepress@plos.org.

You will receive an invoice from PLOS for your publication fee after your manuscript has reached the completed accept phase. If you receive an email requesting payment before acceptance or for any other service, this may be a phishing scheme. Learn how to identify phishing emails and protect your accounts at https://explore.plos.org/phishing.

If we can help with anything else, please email us at customercare@plos.org.

Thank you for submitting your work to PLOS One and supporting open access.

Kind regards,

PLOS One Editorial Office Staff

on behalf of

Dr. Iman Aghayan

Academic Editor

PLOS One
